# Cardiac-Adaptive Conductive Hydrogel Patch Enabling Construction of Mechanical–Electrical Anisotropic Microenvironment for Heart Repair

**DOI:** 10.34133/research.0161

**Published:** 2023-06-08

**Authors:** Xiaoping Song, Jifeng Zhang, Si Shen, Dan Liu, Jie Zhang, Wenming Yin, Genlan Ye, Leyu Wang, Liu Cai, Honghao Hou, Xiaozhong Qiu

**Affiliations:** ^1^ Central Laboratory, The Fifth Affiliated Hospital, Southern Medical University, Guangzhou, Guangdong 510910, China.; ^2^ Department of Anatomy, Neuroscience Laboratory for Cognitive and Developmental Disorders, Medical College of Jinan University, Guangzhou 510630, China.; ^3^ Guangdong Provincial Key Laboratory of Construction and Detection in Tissue Engineering, School of Basic Medical Science; Biomaterials Research Center, School of Biomedical Engineering, Southern Medical University, Guangzhou, Guangdong 510515, China.

## Abstract

The biomimetic construction of a microstructural–mechanical–electrical anisotropic microenvironment adaptive to the native cardiac tissue is essential to repair myocardial infarction (MI). Inspired by the 3D anisotropic characteristic of the natural fish swim bladder (FSB), a novel flexible, anisotropic, and conductive hydrogel was developed for tissue-specific adaptation to the anisotropic structural, conductive, and mechanical features of the native cardiac extracellular matrix. The results revealed that the originally stiff, homogeneous FSB film was tailored to a highly flexible anisotropic hydrogel, enabling its potential as a functional engineered cardiac patch (ECP). In vitro and in vivo experiments demonstrated the enhanced electrophysiological activity, maturation, elongation, and orientation of cardiomyocytes (CMs), and marked MI repair performance with reduced CM apoptosis and myocardial fibrosis, thereby promoting cell retention, myogenesis, and vascularization, as well as improving electrical integration. Our findings offer a potential strategy for functional ECP and provides a novel strategy to bionically simulate the complex cardiac repair environment.

## Introduction

Myocardial infarction (MI) is a leading cause of morbidity and mortality worldwide [1,2]. Following MI, a large number of cardiomyocytes (CMs) are lost due to insufficient blood supply. As a consequence, post-infarction ventricular remodeling begins, where a highly anisotropic myocardium is replaced by a disordered fibrotic tissue because of the limited regenerative potential of the heart and irreversible maladaptive cardiac remodeling [3]. Due to the shortage in heart donors and the limited retention as well as engraftment of cell therapies, tissue engineering approaches have been widely proven to represent a promising alternative to accomplish regenerative repair following heart injury [3–5]. Engineered cardiac patches (ECPs) have been developed to ameliorate post-infarction ventricular remodeling and restore cardiac function by providing mechanical support and an electrically coupled microenvironment [3–6]. To this end, considerable work has been aimed at simulating and reestablishing the mechanical and electrophysiological microenvironment of the extracellular matrix (ECM) of natural heart tissue [3–5,7–10]. However, natural heart tissue consists of a well-ordered and aligned ECM at the micro- as well as nanoscale, and inherently exhibits anisotropic microstructural, mechanical, and conductive properties [7,11]. Surrounding these specific characteristics in the native cardiac microenvironment, living cells in host heart tissues possess high sensitivity and responsiveness to local topographic and mechanical emicroenvironments are critical for not only regulating cell signaling, adhesion, growth, functionalization, and other behaviors but also impacting MI repair performance [11]. Therefore, the development of tissue-adaptive ECP scaffolds to mimic the native ECM microenvironment with anisotropic microstructural, mechanical, and electrical features is vital for MI repair. Recently, various well-designed experiments and synthetic materials, such as 3-dimensional (3D) printing, electrospun nanofibers, and pre-patterning, have been used to simulate the 3D aligned microstructures of natural ECM in cardiac tissues [12–14]. Improved regulation of the macro- and nanoscaole microstructures and biomechanics of biomaterials significantly promotes the potential of these resultant biomaterials for the injury repair, and regeneration of anisotropic cardiac tissues is significantly promoted [15–18].

Despite the great progress in the field of advanced synthetic scaffolds and preparation strategies, several issues, including inadequate biocompatibility, insufficient cell migration, and shortage of ECM-mimicking 3D cell microenvironment to efficiently promote post-infarction cardiac regeneration, still remain to be resolved prior to their use in clinical practice. Although numerous synthetic materials have been prepared to construct functional cardiac scaffolds, additional studies are required to explore the material–cell interactions and seek adequate biocompatibility for increased cell engraftment and therapeutic benefits [11,12]. Compared with synthetic materials, natural biomaterials, fully or partially derived from natural sources, are appealing owing to their perfect biocompatibility with tissues and easy degradability; thus, they have been widely used in tissue engineering [19–21]. Efforts are also being made to construct functional engineered myocardial tissues using natural ECM-derived materials (collagen, fibrin, alginate, and/or Matrigel) [22–24]. Considering that collagen is the most abundant and dominant component (approximately 80% in myocardial tissue) in almost all ECMs, collagen-based biomaterials have received increasing attention in cardiac tissue engineering due to their obvious regulation and promotion effect on cardiac cells and tissue injury repair [25],[26]. Nevertheless, the ability of existing approaches for constructing microstructural scaffolds to mimicking 3D cells and tissue microenvironment is limited, thus hindering the potential for efficient promotion of post-infarction cardiac regeneration. A cardiac-adaptive ECM-mimicking microenvironment with anisotropic microstructural, mechanical, and electrical features is responsible for 3D cell assembly and neo-tissue regeneration for MI repair. Hence, therapeutic performance is expected to be gained by further exploring the integrated design of bioactive, architectural, biomechanical, and electrophysiological properties of scaffold material involved in cardiac tissue constructs.

**Fig. 1.**
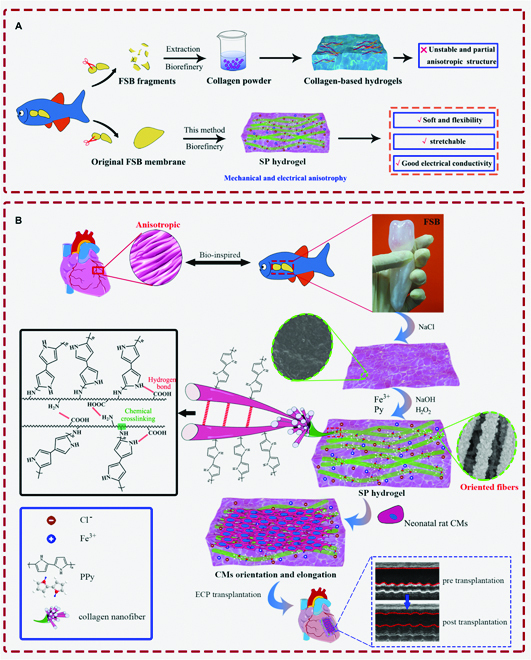
Design of SP hydrogel to recapitulate the anisotropic structure of elongated myofibers for the engineered cardiac patch. (A) Graphic illustration of the method used for preparing the highly flexible, anisotropic, and conductive hydrogel with a relevant 3D spatial structural integrity. We display the transformation from the originally stiff, unstretchable, and nonconductive fish swim bladder (FSB) film and compare our method to the traditional collagen powder extracting–doping–assembly approach. The advantages of our elegant and simple method (lower) compared to the previous study (upper) [31,33], making the originally stiff, unstretchable, and nonconductive FSB film convert to a hydrogel (SSH) with high flexibility, mechanical and microstructure anisotropy, along with conductivity while fully preserving the 3D spatial structure of the FSB films. (B) Schematic illustration of the fabrication of the anisotropic SSH/PPy (SP) hydrogel and the SP hydrogel-derived highly flexible, anisotropic, and conductive cardiac patches meant for MI treatment through myocardial tissue engineering.

The fish swim bladder (FSB), an organ that assists fish in floating freely in the water, ~80% of which is composed of type I collagen fibers, is a promising animal-derived collagen biomaterial from natural aquatic products because of its abundance, cost-effectiveness, as well as low antigenicity and allergenicity [27,28]. Further, type I collagen fibers are also the most abundant in the native myocardium, making up 85% of the total collagen [29]. Studies have shown that the FSB membrane, being characterized by a globally ordered arrangement of collagen fibers, possesses a highly similar matrix composition and hierarchical microstructure to that of natural tissues [27,28,30]. These naturally sophisticated anisotropic microstructures with good biocompatibility in swim bladders make them promising candidates for oriented tissue scaffolds with ordered collagen fiber microstructures. However, the original forms of FSB films are hard and unstretchable and have poor conductivity. As the natural ECM environment of these soft tissues is elastic and conductive, the properties of the original FSB films do not match with the mechanical properties of natural heart ECM tissues and impose restrictions on their application in soft tissue engineering. Therefore, most researchers usually focus on developing a series of approaches to accomplish collagen extraction from various resources and reassemble them in hydrogel matrices for collagen-based scaffolds and tissue engineering (Fig. 1A) [31–33]. However, these extraction processes completely ruin the inherent anisotropy endowed by the ordered collagen fiber microstructures inside FSB materials. Furthermore, these sophisticated ordered microstructures in the swim bladder, the structure of which resembles the typical striped muscles of the natural cardiac tissue, might be better suited for application in cardiac repair. Therefore, the key issue is to convert the originally stiff and rigid FSB membrane film into a flexible and elastic hydrogel with structural similarity to cardiac tissues. It is desirable to develop a feasible and robust method to construct a novel highly flexible, anisotropic, and conductive hydrogel ECP patch with globally ordered collagen fibers that mimic the composition and microstructure of natural cardiac tissues. To the best of our knowledge, limited research has been conducted in this regard.

Inspired by the sophisticated ordered microstructures with anisotropic properties found in a natural swim bladder, we developed a novel nanocomposite hydrogel with high flexibility, anisotropic order, conductivity, and mechanical as well as structural similarities to soft heart tissues based on the originally unstretchable and nonconductive FSB film (Fig. 1). First, we produced a highly anisotropic porous aquatic-derived hydrogel biorefined from a stiff, homogeneous FSB membrane. Through in situ polymerization of conductive polypyrrole (PPy) in the hydrogel and dynamic binding between them, we converted the anisotropic porous hydrogel into a flexible and elastic electroconductive hydrogel. More importantly, using this technique, the anisotropic microstructure of FSB was basically preserved, which is crucial to mimic the cardiac tissue-specific ECM-like architecture. The resultant hydrogel was endowed with high flexibility, mechanical and microstructural anisotropy, and ionic conductivity, which enable it to promote the cellular viability, electrophysiological activity, maturation, elongation, and orientation of CMs. Further in vivo MI rat models have further shown that the developed hydrogel ECP can accelerate exogenous cell retention and vascularization, as well as improve electrical propagation and integration. We believe that this bioinspired engineered patch strategy that integrates high architectural control with mechanical–electrical anisotropy is promising for cardiac tissue engineering applications. In addition, this biorefining approach could substantially contribute to biological medicine, considering the application of renewable feedstocks and green synthetic routes to prepare new materials.

## Results and Discussion

### Design and preparation of ECM-mimicking hydrogel scaffold (SP hydrogel) with mechanical–electrical anisotropy

**Fig. 2.**
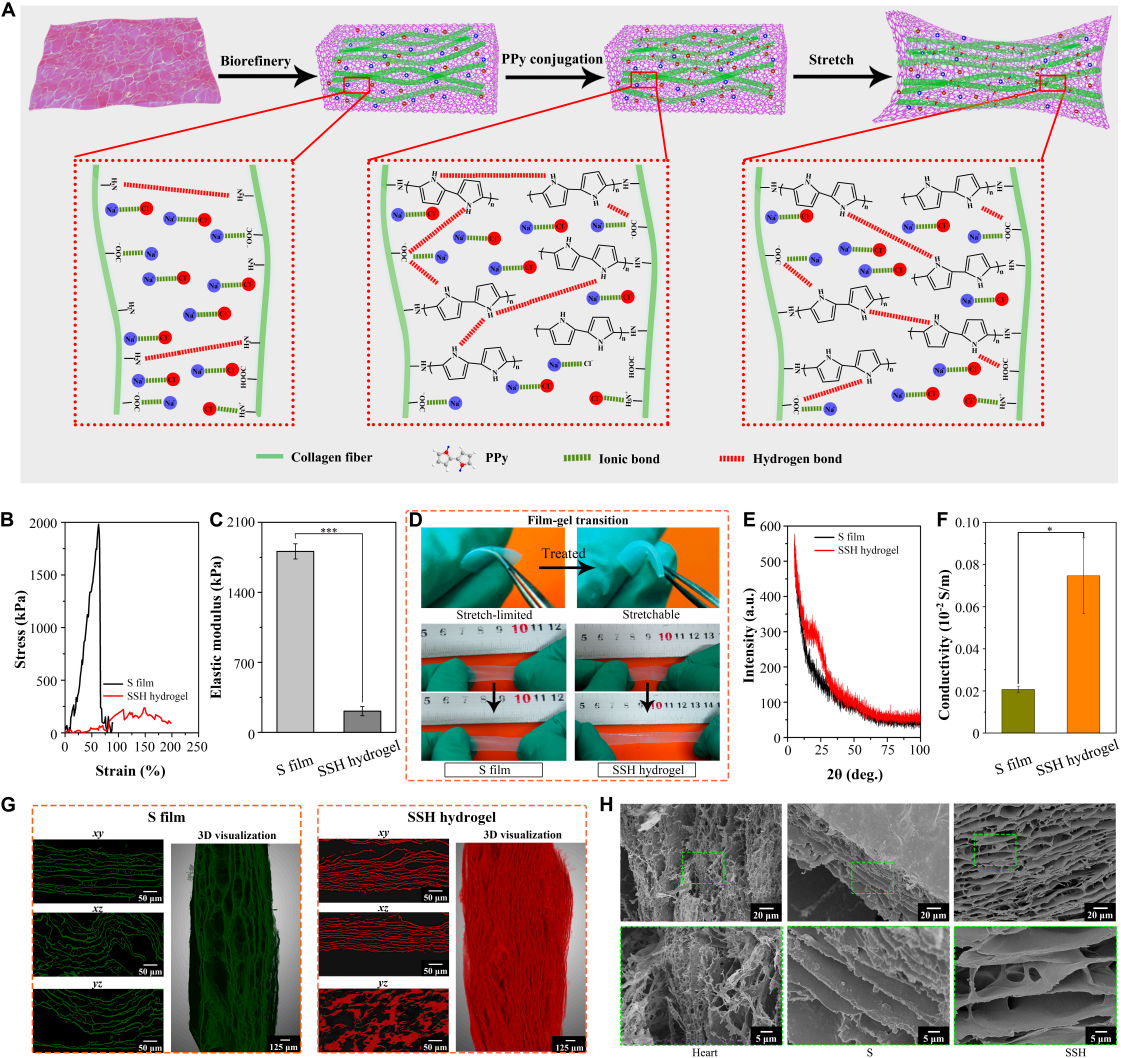
The FSB-derived anisotropic hydrogel demonstrates desired mechanical–conductive properties and anisotropic well-ordered microstructures. (A) Mechanism through which the SSH and SP hydrogel obtained good stretchability. (B) Tensile stress–strain curves of the S film and the SSH hydrogel. (C) Elastic modulus of the S film and the SSH hydrogel. *n* = 5. ***P < 0.001. (D) The treated SSH hydrogel is more flexible and stretchable than the S film. (E) X-ray diffraction (XRD) patterns. (F) Conductivity of the S film and that of our SSH hydrogel. *n* = 5. *P < 0.05. (G) 3D XRM analysis of Sc-treated FSB-derived film (S film) and sodium chloride/sodium hydroxide/hydrogen peroxide (Sc/Sh/Hp)-treated FSB-derived hydrogel (SSH hydrogel). The left 3 images were obtained by scanning the different axis (*xy*, *xz*, and *yz*), and the one on the right is a 3D reconstruction of the entire segment. (H) SEM observation of the different scaffolds under the longitudinal section.

Recently, oxidants or strong bases have been increasingly used for the decellularization of collagen-rich tissues, which is more cost-effective and has also been shown to be effective in disrupting cell membranes and in lysing intracellular organelles [34]. In addition, NaOH treatment can effectively decellularize the tissue and preserve its network structure [35]. Here, a 3D anisotropic hydrogel cardiac patch with a natural ECM-like architecture was created based on the globally collagenous fiber microstructure of the FSB film (Fig. 1B). We first obtained a pristine FSB film crude (S) extract from fresh fish using sodium chloride solution (2.5%, m/v). The FSB film is a solid opaque membrane with oyster white color due to the presence of considerable lipids and proteins. Then, a modified alkaline pre-hydrolysis and peroxide oxidation combinatorial biorefining method [sodium chloride/sodium hydroxide/hydrogen peroxide (Sc/Sh/Hp)] was used to remove immunogenic constituents, such as lipids, sugars, and proteins. Therefore, a highly anisotropic hydrogel [Sc/Sh/Hp-treated hydrogel (SSH)] was directly acquired through the exposure of the hierarchically ordered collagen fibers to Sc/Sh/Hp, distinctly prevailing over previous methods [31–33]. There was almost no immunogenic cellular residue in the obtained SSH hydrogel, which was confirmed through 4′,6-diamidino-2-phenylindole (DAPI) staining and DNA content detection (Fig. S1). Along with the introduction of substantial intermolecular interactions, the obtained SSH hydrogel underwent a transparent transition from a stiff and unstretchable state (original S film) to a stretchable and elastic hydrogel state (Fig. 2A). After the addition of PPy, the obtained SP hydrogels exhibited the desired mechanical properties. This might be due to PPy addition, which strengthened the intermolecular hydrogen bonding force and ensured that the gel network was intact during the stretching process (Fig. 2A). Although it is easily detached from the original binding site after stretching, the detached PPy quickly rebinds to another site, ensuring that the macroscopic properties of the scaffold are not significantly affected. As shown in Fig. 2B and C, the elastic modulus sharply decreased from 1,800 kPa for the original S film to 200 kPa for the SSH hydrogel, which falls within the modulus range of natural cardiac tissue [36]. The obtained SSH hydrogel exhibits higher flexibility and stretchability than the original S film (Fig. 2D). The x-ray diffraction (XRD) patterns indicated that a broad diffused peak at approximately 25° was attributed to collagen fibers, whereas the sharp morphology implied perfect crystallization in the treated SSH hydrogels (Fig. 2E). Notably, compared with the untreated S film, the conductivity of SSH hydrogels has been significantly improved more than 3-fold (Fig. 2F) by introducing numerous ions into them, comparable to the reported ionically conductive hydrogel [37].

**Fig. 3.**
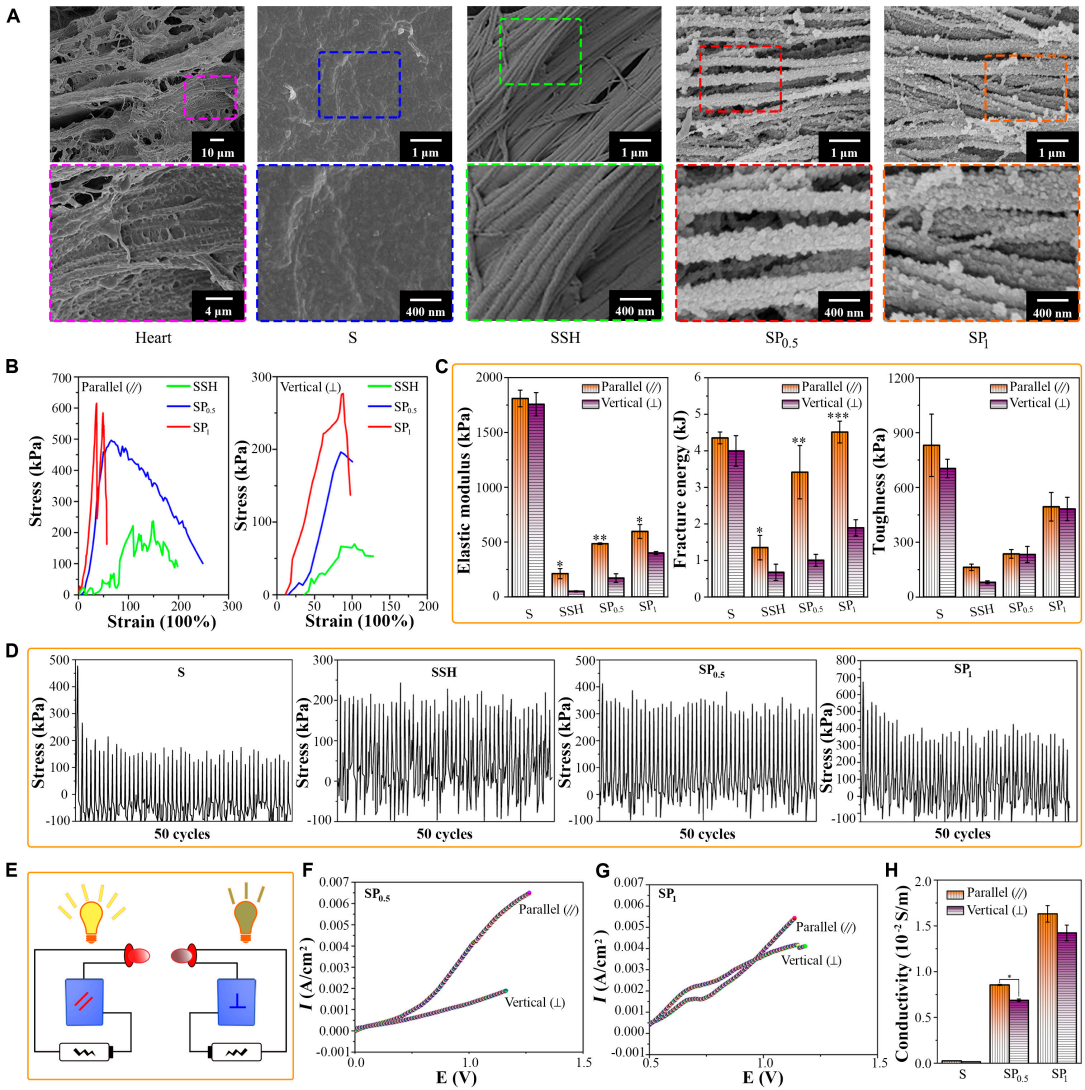
Anisotropic mechanical and electrical properties of the SP hydrogel. (A) SEM observation on the surface of the different scaffolds, including the S film, SSH hydrogel, and SP hydrogels (SP_0.5_ and SP_1_). The morphologies of the SSH and SP hydrogels resemble those of the natural mammalian hearts. (B) Typical tensile stress–strain curves of different scaffolds in the directions parallel and perpendicular to the alignment fibers. (C) Analysis of mechanical property parameters elastic modulus, fracture energy, and tensile strength. *n* = 5. *P < 0.05, compared to that perpendicular to the collagen fibers; **P < 0.01, compared to that perpendicular to the collagen fibers; ***P < 0.001, compared to that perpendicular to the collagen fibers. (D) Cyclic tensile tests of the different scaffolds, including the S film, SSH hydrogel, and SP hydrogels (SP_0.5_ and SP_1_). (E) Schematic diagram for the circuit used to detect the conductive anisotropy of the SP-derived scaffolds. (F) *I*–*V* curves of the SP_0.5_ hydrogel in different directions. (G) *I*–*V* curves of the SP_1_ hydrogel in different directions. (H) Conductivity of the different scaffolds in both parallel and perpendicular directions. *n* = 5. *P < 0.05.

To observe the microstructures of the obtained SSH hydrogel in detail, 3D high-resolution x-ray tomography microscope (XRM) and scanning electron microscope (SEM) analyses were conducted to characterize the detailed structural features of this hydrogel (Figs. 2G and H and 3A and Movies S1 and S2). From the 3D XRM analysis illustrated in Fig. 2G, it is evident that the collagen fibers in the treated SSH hydrogel showed obvious orientation along the *x*, *y*, and *z* axes, thus suggesting a globally ordered microstructure of the hydrogel. As shown in Fig. 2H, the SEM results from the longitudinal section indicated that a well-aligned nanofiber network was formed after a series of treatments, wherein a 3D interconnected lamella-like micronetwork with aligned pores was generated. After treatment with sodium hydroxide and hydrogen peroxide, the collagenous fibers in the SSH hydrogel became relatively looser and more orderly compared to the pure S film, and its organization exhibited some similarities to the microstructure of the natural cardiac ECM. Detailed observation of the FSB hydrogel surface using SEM revealed that the ordered collagenous fibers were exposed in the SSH hydrogel, while excess stromal material was removed from the FSB film after a series of treatments (Fig. 3A). As demonstrated by the aforementioned results, the originally stiff and unstretchable rough FSB film significantly changed and the obtained SSH hydrogel was endowed with adequate elasticity and flexibility while guaranteeing the integrity of its microstructure.

Furthermore, an anisotropic ordered and conductive SSH/PPy hydrogel (SP hydrogel) was obtained through in situ polymerization and covalent cross-linking of pyrrole (Py) monomers at different concentrations onto the collagen-abundant matrix in the SSH hydrogel using FeCl_3_.6H_2_O. The prepared SSH hydrogels treated with different concentrations of Py (0.5 or 1 mg/ml) were named SP_0.5_ and SP_1_, respectively. By coupling a certain amount of PPy in the resultant SP hydrogel, more interaction sites and abundant electrically conductive media, such as various ions and van der Waals forces, were introduced, endowing the SP hydrogel with high conductivity and proper elasticity. Polymerized PPy was uniformly distributed along the direction of the nanofiber after cross-linking with the collagen fibrous network and then assembled into a favorably well-arranged conductive network (Fig. 3A).

We analyzed the Fourier transform infrared spectroscopy (FT-IR) spectra of scaffolds to confirm their chemical composition (Fig. S2). The characteristic absorption bands of collagen were observed at 3,378 cm^−1^ (-OH), 1,655 cm^−1^ (ν_C=O_, amide I band), 1,543 cm^−1^ (δ_N-H_, amide II band), and 1,238 cm^−1^ (ν_C-N_, amide III band). After in situ polymerization of Py monomers onto the collagen fiber matrix, characteristic absorption bands of the Py ring were observed at 1,454 cm^−1^ (ν_C-N_ in the ring), 1,170 cm^−1^ (ν_C-H_ in the ring), and 1,036 cm^−1^ (δ_N-H_ in the ring). With the introduction of PPy, the peaks at 3,378 cm^−1^ (-OH) and 1,238 cm^−1^ (ν_C-N_) in the collagen segment decreased, whereas the characteristic peak assigned to the Py ring increased, demonstrating the successful polymerization of PPy.

The thermal stability and degradation behavior of the S, SP_0.5_, and SP_1_ hydrogels were analyzed using differential scanning calorimetry (DSC) and thermogravimetric analysis (TGA). The DSC thermograms showed that there was a slight reduction in melting temperature of the SP scaffolds (102.5 °C in SP_0.5_, 102.2 °C in SP_1_) compared to that of the S scaffold (109.9 °C) (Fig. S3). This suggests good thermal stability of these SP hydrogel scaffolds. A deep exothermic peak was observed for the pure S scaffold, indicating that significant denaturation occurred inside this material. TGA experiments were conducted to further monitor the thermal stability and chemical composition of samples (Fig. S4). The weight loss in the first step, below 200 °C, should be attributed to the loss of residual moisture. The second step started at 250 °C, which corresponded to the polymer decomposition. After this step, the residual quality of S, SP_0.5_, and SP_1_ was 25.38%, 30.94%, and 30.95%, respectively, indicating slightly higher thermal stability of the SP hydrogel scaffolds than that of the pristine S group. As shown in Fig. S5, each group of scaffolds has a high degradation rate after incubation in PBS for 28 days, which was 70.41 ± 5.93% in S, 60.68 ± 12.32% in SP_0.5_, and 62.28 ± 3.15% in SP_1_ separately, indicating that all of these scaffolds exhibited desirable degradation performances in vitro. The physicochemical stability of the scaffolds was also evaluated by an in vitro collagenase degradation assay (Fig. S6). After 7 days of incubation in collagenase solution, the degree of degradation was greater in group S (80.422 ± 6.382%) compared to SP_0.5_ (59.35 ± 2.20%) and SP_1_ (55.48 ± 1.68%). The results have shown that the biorefined conductive FSB scaffold has a certain degree of resistance to enzymatic digestion, which contributes to its continuous mechanical support for the infarcted sites after implantation.

In addition, the swelling characteristics of the FSB-derived scaffolds were examined using a typical swelling test (Fig. S7). The equilibrated swelling ratio of the FSB film (S) and the consequent SP groups (SP_0.5_ and SP_1_) reached over 300%, demonstrating a good water-retention capability. Interestingly, the developed SP_0.5_ hydrogels reached an equilibrated swelling ratio after soaking in PBS for 1.5 h and could swell sharply (SR > 6), which is mainly attributed to the loose porous structure formed inside the hydrogels after a series of treatments, further substantiating the formation of the 3D hydrogel network.

### Anisotropic mechanical and conductive properties of the SP hydrogel

Owing to the inhomogeneous microstructure within the gel, the mechanical and conductive properties of the SP hydrogel, in both the horizontal and vertical directions, were explored in detail. Both the SSH and the SP (SP_0.5_ and SP_1_) hydrogels exhibited anisotropic mechanical properties due to the aligned nanofibers reinforcing the hydrogel in a uniaxial direction (Fig. 3B and C). They exhibited relatively stiff behavior when the tensile load was parallel (//) to the aligned fibers, compared to that in the cases when the tensile load was perpendicular (⊥) to the collagenous fibers. Additionally, a higher maximum extension was obtained in the parallel direction. For the SP hydrogels, improved resilience was associated with SP_0.5_ (in the 2 directions) compared to that of SP_1_, which might be due to the excessive PPy modifications loaded onto SP_1_, rendering it brittle and fragile. When the tension was parallel (//) to the aligned fiber in the SP, a higher elastic modulus and fracture strength were obtained than those obtained when the tension was perpendicular (⊥) to the fibers. Both the elastic modulus and fracture strength gradually increased with the Py content (Fig. 3C). To achieve promising effects in applications of regenerative medicine and tissue engineering, the inherent elasticity of materials is an important factor to be considered. As shown in Fig. 3C, Young’s moduli of the obtained 3 types of hydrogels (SSH, SP_0.5_, and SP_1_) were 212 ± 46.30 kPa, 485 ± 7.07 kPa, and 596.67 ± 63.51 kPa, respectively, in the parallel direction. Alternatively, the 3 types of scaffolds had a modulus of 51 ± 5.06 kPa, 171.25 ± 39.43 kPa, and 400 ± 14.14 kPa, respectively, in the perpendicular direction (Fig. 3C). These values of the 3 groups are approximately similar to those of mammalian myocardial tissue (20 to 500 kPa) [36] and consistent with the elastic modulus described in previous studies [3,35,36,38,39] (Fig. S8). However, for the hard film (S), the elastic modulus in different directions was beyond that associated with the optimal range for the myocardium. After 50 repeated tensile cycles on SSH and SP_0.5_, there was almost no obvious stress loss, indicating its robust flexibility and elasticity (Fig. 3D). In contrast, the S and SP_1_ groups exhibited a relative decrease in the stress loss over 50 cycles.

After oxidative polymerization in the presence of FeCl_3_, conductive PPy was synthesized in situ and cross-linked on the collagen fibers of the SSH hydrogel. Further, numerous Na^+^ and Cl^−^ ions were introduced, thus endowing the SP hydrogel with excellent flexibility and electrical conductivity (Fig. 3E to H). The anisotropic nature of the SP hydrogel with oriented fibers is reflected in its electrical properties. For example, in the direction parallel to the fibers, the SP hydrogels had conductivities of 0.855 ± 0.006 × 10^−2^ S/m (SP_0.5_) and 1.631 ± 0.090 × 10^−2^ S/m (SP_1_), while conductivities of 0.687 ± 0.015 × 10^−2^ S/m (SP_0.5_) and 1.423 ± 0.086 × 10^−2^ S/m (SP_1_) were obtained in the perpendicular direction (Fig. 3H).

### Anisotropic SP hydrogel facilitated CM elongation, orientation, and maturation in vitro

**Fig. 4.**
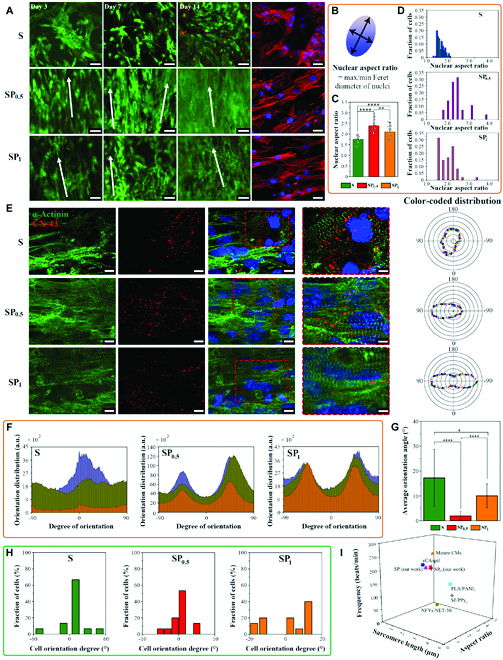
The apparent elongation, alignment, and maturation of primary CMs induced by the anisotropic well-ordered SP hydrogels. (A) Columns 1 to 3: Live/dead staining of cells cultured on 3 types of scaffolds from day 3 to day 14. Column 4: CMs on the SP hydrogels display elongated and oriented morphologies, with F-actin stained using rhodamine–phalloidin (red) and nuclei stained using DAPI (blue). Scale bars, 50 μm (columns 1 to 3) and 25 μm (column 4). (B) Cell elongation was estimated using the nucleus aspect ratio (maximum/minimum Feret diameter). (C) The statistical analysis showed that the nucleus aspect ratio of cells cultured on the SP hydrogels was significantly higher than that of those cultured on the S film on day 7. **P < 0.01; ****P < 0.0001. (D) The distribution analysis of the nucleus aspect ratio demonstrated well cellular elongation within the SP hydrogels (especially in SP_0.5_). (E) Fluorescence microscopy images showing the levels of the sarcomeric α-actinin proteins (green) and connexin-43 proteins (CX-43, red) in the CMs seeded on the different hydrogel scaffolds on day 7. Column 5: radar graph analysis about orientation distribution of CMs. Scale bars, 10 μm (columns 1 to 3) and 5 μm (column 4). (F) Curve analyses of orientation distribution of CMs from 3 individual images in the different scaffolds. (G) Average orientation angles for CMs in the different scaffolds. *n* = 10. *P < 0.05; ****P < 0.0001. (H) Distribution of orientation angles for cells in the different scaffolds. (I) Comparison of sarcomere length, aspect ratio, and beating frequency of recently described conductive scaffolds and our SP scaffolds.

It is crucial to construct an aligned microenvironment that mimics the anisotropic structure of natural cardiac tissues [40]. Scaffolds composed of aligned morphology can provide topographical cues to induce CM alignment and elongation [11,15]. To test the impact of the aligned architectural cues on the viability, alignment, and elongation morphologies of CMs, ECPs were fabricated by seeding neonatal rat CMs on the different scaffolds (S, SP_0.5_, and SP_1_). All ECP groups exhibited excellent cell compatibility (Fig. 4A and Movie S3), and the CMs showed different degrees of elongation in the 3 groups, which was particularly evident in the SP_0.5_ group. CMs on the conductive SP scaffolds formed an obviously dense, orientated myocardium after culture for 14 and 21 days (Fig. 4A and Fig. S9). Furthermore, an intact cardiac syncytium-like structure was formed between the elongated cardiac cells and the 3D matrix frame in the SP-ECPs (SP_0.5_ and SP_1_), as observed using SEM (Fig. S10), thus suggesting that our prepared anisotropic SP hydrogel scaffolds had extremely fine biocompatibility and induced cell alignment. Notably, unlike conductive SP hydrogels (SP_0.5_ and SP_1_), flat cells were tiled on top of nonconductive FSB membranes (S), showing a typically irregular arrangement. To assess the biocompatibility of the scaffolds for various types of cells, the cells including human CM cell line H9C2, cardiac fibroblast, and human endothelial cell line HUVEC were cocultured with the scaffold (Figs. S11 to S13). The results have shown that our developed FSB hydrogel could serve as a promising cardiac patch candidate for various types of cells.

Cytoskeletal fluorescent images using F-actin staining showed that elongated CMs were distributed uniformly onto SP hydrogels after 3 days (Fig. 4A) and 7 days (Fig. S14 and Movie S4), whereas cells on the pure FSB film (S group) exhibited a random morphology. This indicates that the conductive SP scaffolds could provide a suitable environment for guiding the elongation and alignment of CMs via their oriented topological and electrophysiological cues. In particular, the SP_0.5_ hydrogel showed a more remarkably enhanced cardiac cell orientation compared to SP_1_. This difference may be explained by the excessive PPy in SP_1_, which might have weakened or even eliminated the microstructural and conductive anisotropic properties of the resulting hydrogel, along the vertical and horizontal directions. In detail, part of the PPy in the SP_1_ group was introduced along the collagen fibers, and the other part was gathered between collagen nanofibers (SEM observation). As illustrated in the *I*–*V* curve analysis depicted in Fig. 3G, the vertical conductivity has a steeper drop than that of the parallel conductivity at the cross point of the voltage value of approximately 1 V. The existence of a cutoff point ultimately reduced the conductivity anisotropy.

The Feret diameter ratio (nucleus aspect ratio) is a typical method for determining the degree of ellipticity of a contour by calculating the ratio of the maximum to minimum diameter [41]. In highly anisotropic tissues, the Feret diameter ratio varies from 1.8 to 6, whereas in isotropic tissues, it varies from 1.1 to 1.8 [42]. Long-term cell culture on anisotropic scaffolds in vitro may result in elongated nuclei. Moreover, the 3D architecture of host tissues may promote a greater increase in the nucleus aspect ratio [43]. Here, cellular elongation was quantitatively evaluated by the nucleus aspect ratio (Fig. 4B), and our data showed that the nucleus aspect ratios of SP-derived ECPs (SP_0.5_ and SP_1_) were significantly higher than those of S-derived ECP (Fig. 4C). When seeded on the isotropic S scaffold, the cells reached a nuclear aspect ratio of 1.58. On the highly anisotropic SP scaffolds, the nucleus aspect ratio of seeded cells increased to 2.24 (SP_0.5_) and 1.90 (SP_1_), indicating that the seeded cells could sense the anisotropy of the scaffolds and respond accordingly. Specifically, within the range of 1.8 to 4 Feret diameter, approximately 93% (SP_0.5_ group) and 54% (SP_1_ group) of the cells showed significant elongation, whereas only approximately 9% showed elongation in the S group, indicating the prominent ability of the SP scaffold to guide CM elongation (Fig. 4D).

Conductive scaffolds have been previously proven to facilitate CM maturation as well as cell–cell coupling via gap junctions [44]. In this study, we analyzed the protein expression and distribution of maturity-related markers in CMs. Specifically, sarcomeric α-actinin is a protein involved in the actin–myosin contraction complex [45] and connexin-43 (CX-43) is a gap junction protein involved in the electric-contraction coupling of the myocardium, thus triggering the overall beating of the cardiac muscle [46]. On day 7, seeded cardiac cells in all groups, particularly in the SP-derived ECPs, showed abundant cell-to-cell contacts (CX-43-positive coverage area) and organized sarcomeric striations (α-actinin-positive coverage area) (Fig. 4E). Orientation graphs showed that CMs were arranged in a certain direction in the SP hydrogels (especially in SP_0.5_), whereas more CMs were randomly distributed in the S scaffold (Fig. 4E). Directional distribution curves based on different fields also confirmed that CMs in the SP hydrogel grew in a certain direction (Fig. 4F). To evaluate the degree of “contact guidance” of the scaffold, the positioning angle between the cell growth axis and the standard line was measured. The direction parallel to the swim bladder fiber was set as the standard line direction (0°). The cells in the S group had the broadest distribution of orientation angles, whereas those in the SP_0.5_ group had the narrowest distribution (Fig. 4G and H). The average orientation angle of cells in the SP_0.5_ group was the smallest and closest to 0° (1.97°), while that of the S group was relatively large (17.23°). Furthermore, there were relatively small orientation angles in the SP_1_ group (10.04°). These findings indicate that during expansion, the cells were restricted by the anisotropic fiber pattern to the FSB scaffold, forcing them to grow and elongate in the direction of the fibers. Additional parameters associated with CM maturation are shown in Fig. 4I and Fig. S15, wherein the sarcomere length and aspect ratio of CMs in SP_0.5_ are close to that of mature CMs. The comparison concerning the promoting effects of conductive scaffolds [43,47–49] on CM growth reported in recent years revealed that the prepared cardiac-adaptive conductive SP was a good matrix for CM sarcomere elongation and maturation (Fig. 4I). Consequently, the natural FSB-derived SP hydrogels with anisotropic mechanical and electrical characteristics are promising ECP scaffolds that can induce oriented cell adhesion, growth, and maturation.

### Anisotropic SP hydrogel encouraged electrophysiological development and synchronized contraction of CMs

**Fig. 5.**
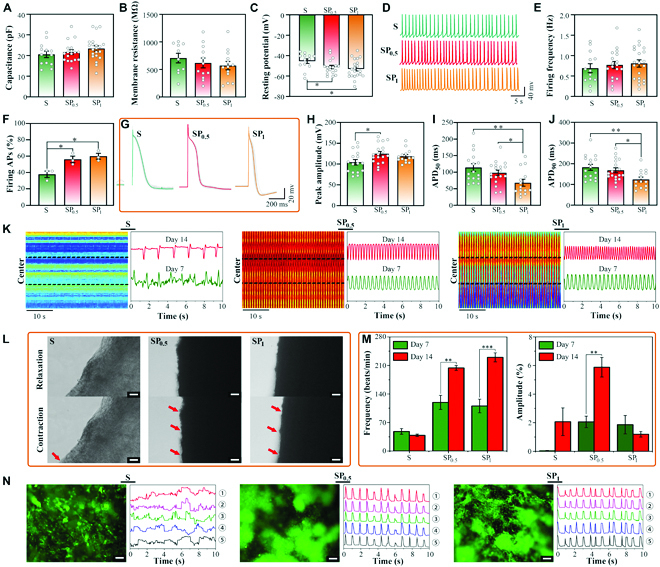
Electrophysiological properties of the CMs seeded on S, SP_0.5_, and SP_1_ separately. (A to C) Statistical analysis of capacitance, input resistance, and resting potential of CMs cultured on S, SP_0.5_, and SP_1_ at 3 days. *P < 0.05. (D) Representative tracings of spontaneous AP of CMs cultured on S, SP_0.5_, and SP_1_. (E) Statistical analysis of firing frequency. (F) More cells cultured on SP_0.5_ and SP_1_ hydrogels are able to spontaneously fire trains of APs than those cultured on the S film. *P < 0.05. (G) Representative tracings of evoked APs. The light gray traces on different groups represent the original recordings of evoked APs; the green, red, and orange traces represent the average curves from light gray traces of *n* = 15 trials on every group, respectively. (H to J) Statistical analysis of peak amplitude of AP, APD_50_, and APD_90_ of CMs cultured on S, SP_0.5_, and SP_1_ scaffolds. *P < 0.05; **P < 0.01. (K) Beating behavior of CMs. Left: Color map in the different hydrogels indicates the relatively more uniform contraction behavior apparent in the SP hydrogels. Right: The beating curves of the different ECPs confirm the more uniform contraction behavior associated with the cells cultured on SP-ECPs on days 7 and 14. (L) Light microscope imaging of the different ECPs during contraction and relaxation at day 7. Scale bars, 50 μm. (M) Spontaneous beating rates and spontaneous beating amplitudes of the different ECPs on days 7 and 14. *n* = 5. **P < 0.01; ***P < 0.001. (N) Related frequency signals within 10 s at 5 individual points extracted from intracellular Ca^2+^ changes in different scaffolds at day 7. *F*/*F*_0_: Measured fluorescence normalized to the background fluorescence. Scale bars, 50 μm.

Electrophysiological development and maturation are essential indicators for evaluating the functions of engineered cardiac tissue models. To explore the effect of the SP-based microstructural–mechanical–electrical anisotropic microenvironment on the electrophysiological maturity of CMs, various relative parameters were introduced to evaluate the electrical activity of CMs grown on S, SP_0.5_, and SP_1_. Electrophysiological measurements were performed using a patch-clamp on a single cardiac cell after 3 days of culture when the single cardiac cell still did not form unfused syncytia. Membrane passive characteristics, such as capacitance, input resistance, and resting membrane potential, are widely recognized indicators for assessing cellular development and health [50]. As shown in Fig. 5, no differences in the passive membrane properties (cell capacitance and input resistance) were observed among these 3 groups (Fig. 5A and B), whereas the resting potential values of CMs grown on SP_0.5_ or SP_1_ were more negative than those of CMs grown on S (Fig. 5C). These results suggest that the conductive SP hydrogel may accelerate the maturation of CMs by affecting the resting membrane potential without significantly altering other measured characteristics.

We further examined the spontaneously generated action potentials (APs) of single cardiac cells cultured on different materials under a current clamp configuration. As shown in Fig. 5D, all CMs cultured on FSB scaffolds could generate spontaneous APs. Regardless of the culture conditions, single CMs generated APs at almost the same frequency (Fig. 5E). Interestingly, the probability of CMs grown on SP_0.5_ and SP_1_ to be able to fire APs was significantly higher than that of CMs grown on S (Fig. 5F), thus indicating that both SP_0.5_ and SP_1_ hydrogel scaffolds can cause more CMs to spontaneously fire APs atop their surface compared to the S group.

We also analyzed the evoked APs of CMs using a current-clamp configuration at −70 mV, and APs were induced by injecting brief (5 ms) square depolarizing current steps (0.5 nA; 0.2 Hz). Figure 5G shows the superimposed traces of evoked APs sampled from CMs grown on S, SP_0.5_, and SP_1_ (the light gray traces on different groups represent the original recordings of evoked APs from the 15 stimulations; the green, red, and orange traces represent the average curves from light gray traces of *n* = 15 trials in every group, respectively). From the analysis results of AP kinetics shown in Fig. 5G and H, it is evident that the peak amplitude of AP was increased in the CMs cultured on SP_0.5_ when compared with that of the other 2 groups, indicating more CM maturation resulting from a more significant promotion endowed by SP_0.5_. In addition, the recordings showed that the AP shape and kinetics of CMs cultured on S and SP_0.5_ were similar to those usually described for mature mammalian CMs and those cultured on other conductive media [51,52]. However, AP shape and kinetics of CMs cultured on SP_1_ were different. The action potential duration (APD) at 50% (APD_50_) and 90% (APD_90_) repolarization of CMs cultured on SP_1_ were significantly shorter than those of CMs cultured on S and SP_0.5_ (Fig. 5I and J). The APD shortening indicates an acceleration of the repolarization process. We hypothesize that the high conductivity of SP_1_ may affect the function of ion channels involved in the repolarization process. Based on these findings, we concluded that SP_0.5_ was more beneficial for CM electrophysiological maturation.

Moreover, other electrophysiological maturation-associated parameters, including beating behavior and calcium transient calcium propagation, were further investigated to monitor the properties of AP-induced spontaneous beating activity in the engineered myocardium. First, the spontaneous beating behavior of the whole cell population was recorded in all the ECPs on day 7 (Fig. 5K and Movie S5). A relatively rhythmic and synchronous beating in SP hydrogel ECPs and a mussy beating in the S ECPs were observed. Nearly uniform contractions were observed in the SP_0.5_ ECP, while a relatively random contraction in most areas was detected in S ECP and SP_1_ ECP (Fig. 5K). Among all the ECPs, the SP hydrogel ECPs displayed an overall beating with a relatively rapid rhythm and large contraction (Fig. 5K to M). The S ECP exhibited irregular contractions on days 7 and 14 at a frequency ranging between 48.00 ± 6.93 and 38.67 ± 3.06 beats/min (bpm; Fig. 5M), while SP ECPs exhibited rhythmic beating on days 7 and 14 with frequencies increasing from 119.33 ± 17.24 bpm to 204.00 ± 6.01 bpm for SP_0.5_, and from 110.67 ± 16.78 bpm to 230.00 ± 11.14 bpm for SP_1_. It is worth noting that although the beating frequency of the SP_1_ ECP was the highest, the beating amplitude was decreasing (Fig. 5M and Movie S6). Additionally, the beating frequency and amplitude of SP_0.5_ ECP are closest to those of mature CMs (Fig. 4I).

Calcium signaling within the seeded CMs was then investigated by recording the changes in intracellular calcium concentration on day 7 using Fluo-4 AM as the calcium indicator (Fig. 5N). A spontaneous increase in Ca^2+^ concentration was observed using the fluorescent intensity of dye (*F*) divided by the background intensity (*F*_0_). After 7 days of culture, CMs grown on the SP hydrogels displayed relatively rhythmic Ca^2+^ fluctuations compared to those cultured on S scaffold (Fig. 5N and Movie S7). When CMs were grown on conductive SP hydrogels, a larger proportion of cardiac cells was able to fire an AP, followed by increased integral Ca^2+^ fluctuations compared to those in S scaffolds, thus suggesting that introducing PPy into the SSH hydrogel accelerated CM maturation. Moreover, compared to the rhythm and frequency of cells grown on SP_1_ hydrogel, those of cells grown on the SP_0.5_ hydrogel were more stable and regular. This may be due to the addition of excess PPy that resulted in aggregation and affected the anisotropy, which in turn limited the potential for aligning the CMs and ultimately affected the ability of the functional myocardial tissue to beat synergistically in vitro. Altogether, these indicated that SP_0.5_ containing aligned PPy and collagen fibrous framework could provide a natural ECM-mimicking microenvironment for effectively enhancing electrical coupling within the planted cells and notably improving the beating characteristics of engineered cardiac tissue in vitro.

### Anisotropic SP hydrogel ECPs enhanced cardiac performance in the MI rats

**Fig. 6.**
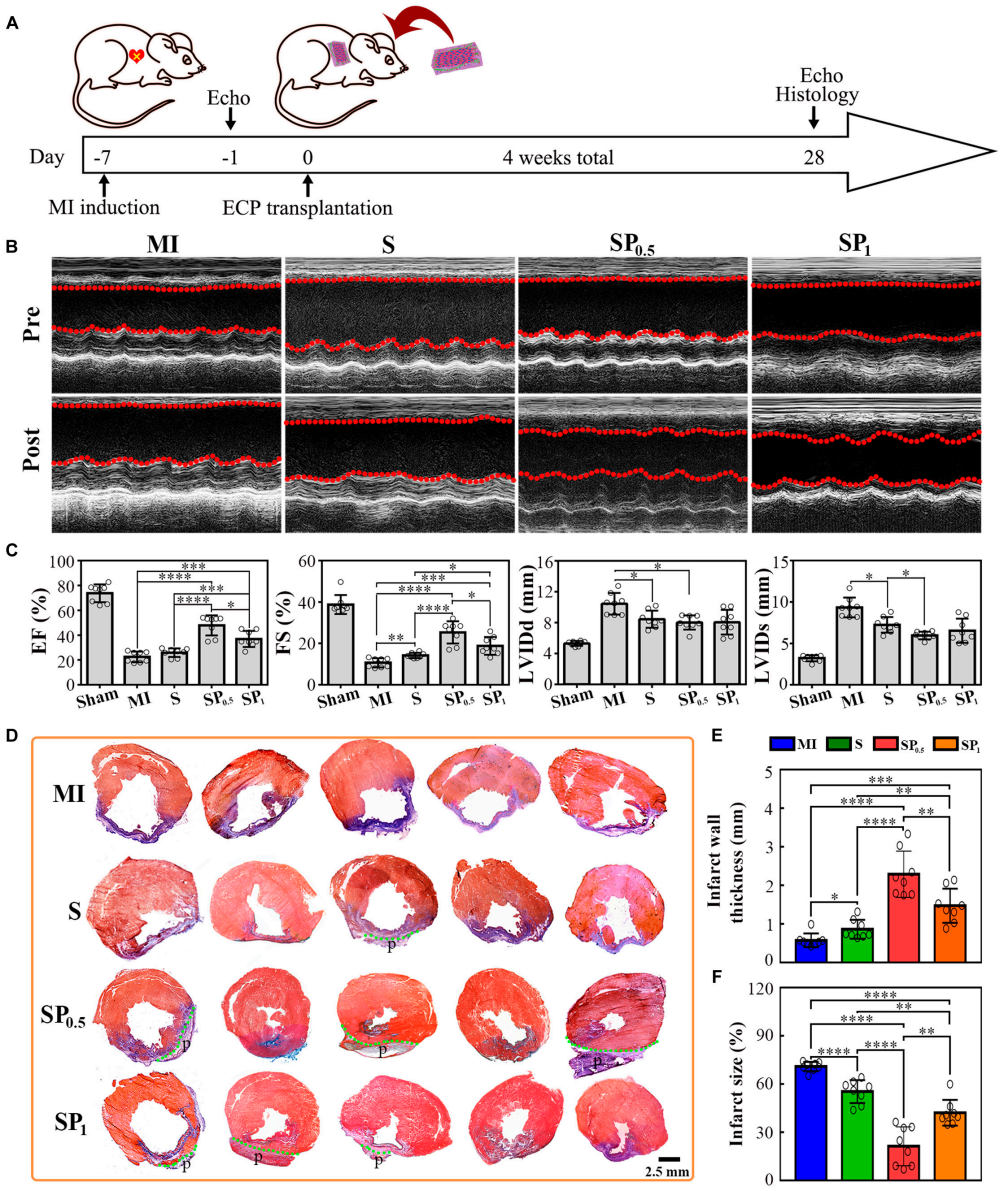
In vivo MI repair effects after implantation of different ECPs in rat MI models for 4 weeks. (A) Schematic representation of the animal experiments conducted for testing the therapeutic benefits of SP hydrogel ECPs in rat MI models. (B) Representative echocardiographic images of hearts before and after transplantation of different ECPs (4 weeks after transplant). (C) Left ventricular (LV) functional parameters (EF, FS, LVIDd, and LVIDs) at 4 weeks after the implantation of different hydrogel ECPs. *n* = 5. *P < 0.05. (D) Representative Masson's trichrome staining for various groups after transplantation at 4 weeks. (E) LV wall thickness in the different hydrogel ECP groups. *n* = 5. *P < 0.05; **P < 0.01; ***P < 0.001; ****P < 0.0001. (F) Fibrosis area in the different hydrogel ECP groups. *n* = 5. **P < 0.01; ****P < 0.0001.

Further, we implanted the SP-derived ECPs into the MI model rats for further in vivo experimental validation (details related to the MI models and ECP transplantation can be found in the Supplementary Materials). The ECPs were transplanted into the MI models 1 week postoperatively. At 4 weeks after the ECP transplantation, we assessed cardiac function using echocardiography in the Sham, MI, S-ECP, SSH-ECP, SP_0.5_-ECP, and SP_1_-ECP groups (Fig. 6A to C and Figs. S16 and S17). Echocardiographic data (Fig. 6B and Movie S8) indicated that the thickness and contractile activity of the free wall in the left ventricle (LV) were obviously ameliorated in the SP-ECP groups. The mean fractional shortening (FS) and ejection fraction (EF) were also significantly improved in the SP-ECP groups compared with those in the MI and S-ECP groups (Fig. 6C). In particular, the mean EF significantly increased in the SP_0.5_-ECP group, indicating that SP_0.5_-ECP was more dominant in maintaining heart function than SP_1_-ECP. In the SSH-ECP group, we observed slight enhancements in LV function parameters (EF = 30.21 ± 2.58%, FS = 16.90 ± 2.18%) compared to those in the MI-ECP (EF = 20.83 ± 5.12%, FS = 10.58 ± 2.25%) and S-ECP groups (EF = 25.58 ± 2.17%, FS = 13.22 ± 0.72%), thus suggesting that the SSH scaffold with excellent 3D structure and a certain extent electrical conductivity exhibits some repairing effects. Nevertheless, SP_0.5_ plays the most obvious role in inhibiting ventricular dilatation. In addition, the repair effect of the ECP group was better than that of the empty scaffold group, indicating that exogenous CMs seeded on the scaffold could also play a crucial role in MI repair (Fig. S18).

After MI, continuous ischemic anoxia causes the death of a large number of CMs, eventually leading to inflammation and diffuse myocardial fibrosis. This in turn induces LV remodeling, including regional LV wall thinning and progressive cavity dilation [44]. Restraining fibrotic remodeling is an effective way to prevent heart failure [37]. Therefore, we further evaluated the intervening effect of transplanted ECPs on myocardial remodeling by analyzing the changes in cardiac fibrosis and LV morphology after ECP transplantation using Masson's trichrome staining (Fig. 6D to F and Figs. S17 to S19). Ventricular wall thinning was observed in the MI and S-ECP group, and almost all normal myocardia (red) were replaced by blue-stained collagen fibers. In the SSH-ECP group, the LV wall was significantly thicker than that in the MI- and S-ECP groups, and a part of the LV free wall was covered by the myocardium (Fig. S16). The fibrotic area decreased sharply, and the ventricular wall thickened in the SP-ECP groups. In particular, the best repair effect was achieved in the SP_0.5_-ECP group, in which the thickness of the LV wall increased from 0.575 mm (at week 4 after MI surgery) to 2.289 mm (SP_0.5_-ECP transplantation for 4 weeks) (Fig. 6E), whereas the infarct size decreased from 70.95% (4 weeks after MI surgery) to 21.24% (SP_0.5_-ECP transplantation for 4 weeks) (Fig. 6F). We also compared the repair effect of the empty scaffold with that of cell-loaded ECP on myocardial remodeling (Fig. S18). The results showed that, compared to the empty scaffold group, the ECP group effectively inhibited fibrosis and promoted ventricular wall thickening. Among these, tissue-adaptive SP_0.5_-ECP exhibited the most significant effects. The electrical activity associated with SP_0.5_-ECP can elevate the retention of the exogenous seeded cells in the infarcted myocardium and augment CM maturation as well as functionality, thus boosting its potential functions in guiding microenvironment reconstruction during MI repair.

### Anisotropic SP hydrogel reduced inflammation as well as apoptosis and promoted electrical coupling and vascularization in the post-MI heart

**Fig. 7.**
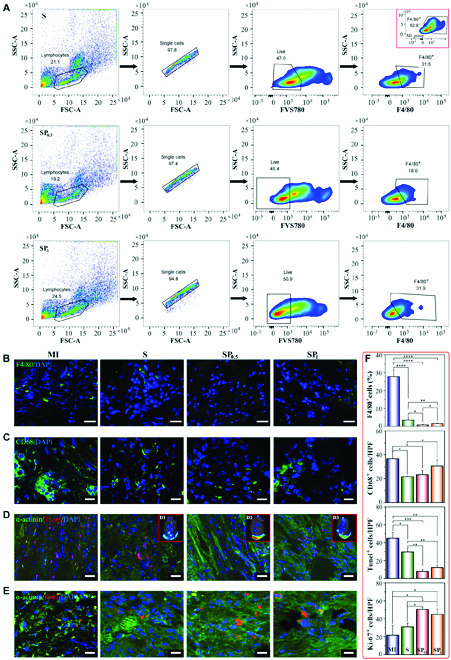
Inflammation alleviation and apoptosis suppression effect of the SP hydrogel ECPs. (A) The flow cytometry results from the isolated cells in the infarcted area 1 week after transplantation indicate that the SP_0.5_ treatment effectively suppressed the inflammatory response. (B) Fluorescent micrographs showing the presence of F4/80^+^ cells (green) in the different groups at 4 weeks after the transplant. Scale bars, 15 μm. (C) Fluorescent micrographs showing the presence of CD68^+^ cells (green) in the different groups at 4 weeks after the transplant. Scale bars, 15 μm. (D) Fluorescent micrographs showing apoptotic (Tunel^+^, red) CMs (α-actinin, green) in the MI hearts at 4 weeks after the transplant. D1 to D3 indicates the status of CM retention in the MI zone after scaffold transplantation at 4 weeks after the transplant under in vivo imaging of small animals. Scale bars, 15 μm. (E) Fluorescent micrographs showing Ki-67-positive (red) and α-actinin-positive (green) cells in the MI hearts at 4 weeks after the transplant. Scale bars, 15 μm. (F) Statistical analysis concerning F4/80^+^ cells, CD68^+^ cells, Tunel^+^ apoptotic cells, and Ki67^+^ cells. *n* = 5. *P < 0.05; **P < 0.01; ***P < 0.001; ****P < 0.0001.

To further explore the underlying mechanism of the SP hydrogel patches for MI repair, we assessed the efficacy of SP patches in alleviating inflammatory stress, repairing electrical coupling, and promoting vessel regeneration between healthy and MI regions (Fig. 7). After implantation for 1 week, the ECPs in each group could effectively inhibit the occurrence of inflammation, especially in the SP_0.5_ group, where the ratio of F4/80^+^ cells was 18.0, while that in the MI group was 62.8 (Fig. 7A and Fig. S20). After 4 weeks of transplantation, no significant increase in F4/80^+^ inflammatory cell infiltration was observed in the ECP-treated hearts (Fig. 7B and F). In addition, the CD68^+^ macrophage densities were significantly decreased across the 3 ECP groups (especially in the S and SP_0.5_ groups) compared to those in the MI group (Fig. 7C and F), thus suggesting that the patches alleviated the inflammation of the MI heart. Besides, the FSB patch transplantation group effectively protected CMs from the microenvironment, and apoptotic CMs were significantly reduced (Fig. 7D and F). The protective effect of the conductive transplant group (especially SP_0.5_) was more obvious than that of the nonconductive group. To track the viability of CMs encapsulated in the scaffolds in real time, we labeled them with the live cell tracer DiI and assessed the fluorescence intensities at different time points using in vivo imaging technology (Fig. 7D1 to D3 and Fig. S21). The results showed that the fluorescence intensity associated with CMs on the conductive SP scaffold was significantly higher than that of CMs in the nonconductive scaffold. Furthermore, there was intense fluorescence in the infarcted area of the SP scaffolds 4 weeks after implantation. CMs adjacent to the MI could be stimulated to enter the cell cycle by the infarct microenvironment after MI [53]. Approximately 4% of myocytes stained positive for Ki-67 in the border zone between damaged and healthy sites [53]. Similar to previously reported data, a small number of proliferating Ki-67-positive cells were detected around the MI sites in our experiments (Fig. 7E and F), while many of them were also positive for the cardiac marker sarcoma α-actinin.

**Fig. 8.**
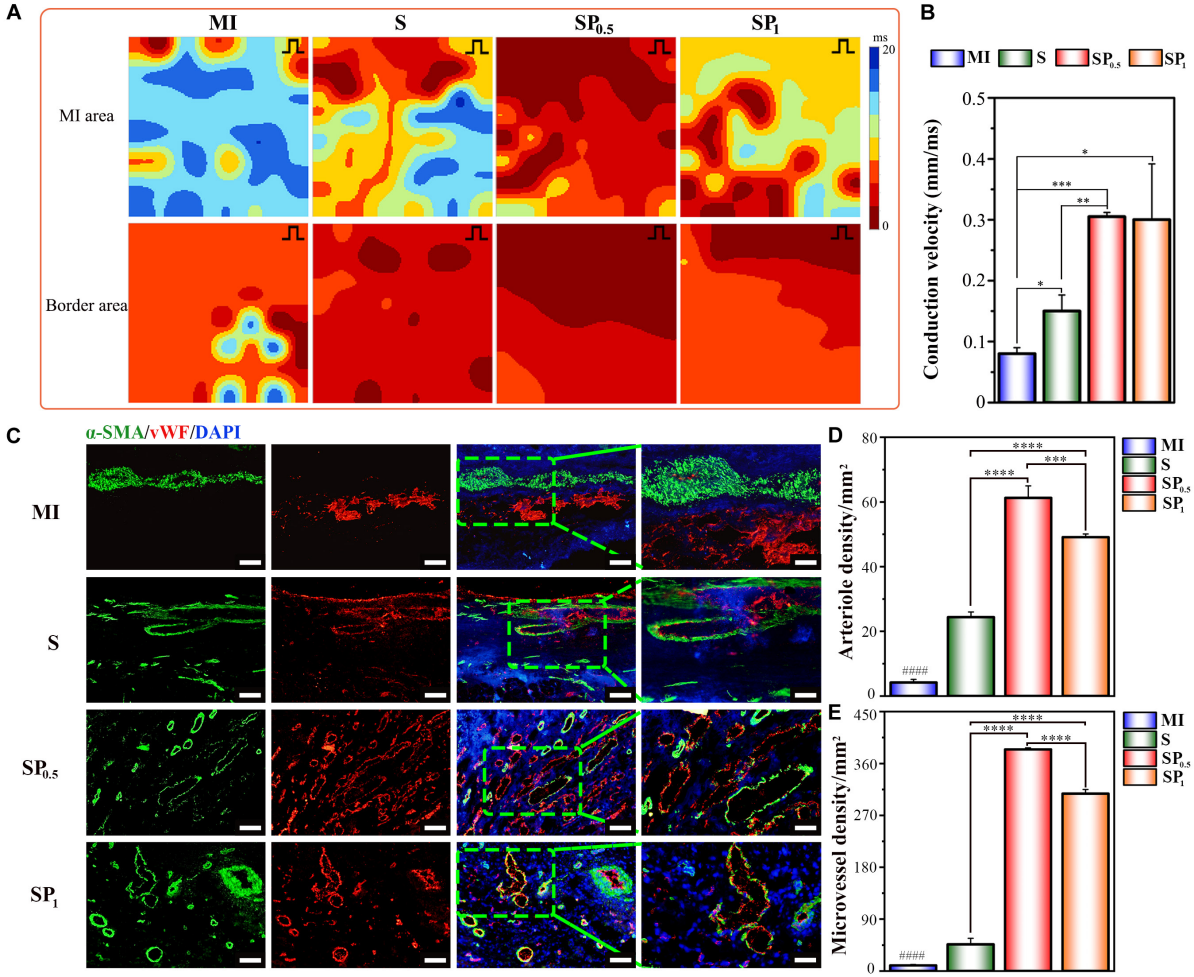
SP hydrogel ECP promoted electrical conduction and revascularization in the infarcted heart. (A) Representative electrical maps of the isolated rat heart in different ECP groups. The earliest activation is exhibited in red, the latest activation is shown in blue, and the numbers indicate the activation time (ms). Upper: The images indicated conduction and propagation of excitation in the infarcted sites. Lower: The images indicated conduction and propagation of excitation between the infarcted and healthy sites. (B) The CV between infarcted and healthy sites is significantly improved in the SP hydrogel ECP groups. *n* = 5. *P < 0.05; **P < 0.01; ***P < 0.001. (C) α-SMA staining (green) and vWF staining (red) of the infarcted areas at 4 weeks after the transplantation of different ECPs. Scale bars, 250 μm (columns 1 to 3) and 100 μm (column 4). (D) Statistical analysis of arteriole densities in different hydrogel ECP groups. *n* = 5. ^####^*P* < 0.0001, compared to MI group. ***P < 0.001; ****P < 0.0001. (E) Statistical analysis of microvessel densities in different hydrogel ECP groups. *n* = 5. ^####^*P* < 0.0001, compared to MI group. ^****^P < 0.0001.

To examine whether SP hydrogel ECPs affected electrical propagation and activity across the ventricle, epicardial mapping of the LV free wall was performed on isolated Langendorff-perfused hearts [54]. Ventricular activation was significantly disturbed and delayed in the MI heart, as shown by the slowed conduction velocity (CV) in MI hearts (Fig. 8A and B and Figs. S22 and S23). In the SP groups (especially SP_0.5_), ventricular activation significantly improved, and the conduction, as well as propagation of excitation between the infarcted and healthy sites increased. This may be due to the suitability of the mechanical–electrical microenvironment in SP_0.5_-ECP that can restore synchronous beating following MI by regulating the transmission of electrical signals within the infarcted area and permitting contraction of viable myocardium insulated by the infiltrated scar tissues [9]. In addition, the CV of the cell-loaded ECP transplantation group was more obvious than that of the empty conductive scaffold transplantation group ex vivo (Fig. S23). This indirectly shows that exogenous CMs seeded on the scaffold are involved in the process of heart repair.

Heart cells are prone to apoptosis caused by MI-induced hypoxia, and angiogenesis can reduce MI-caused damage by mitigating hypoxia [55]. Angiogenesis plays an essential role in maintaining cardiac vascularization and blood perfusion in physiological and pathological host cardiac tissues [55]. Angiogenesis was assessed at the infarcted region in each group by detecting the microvessel [von Willebrand factor (vWF)] and arteriole [vWF and α-smooth muscle actin (α-SMA)] protein-labeled vessels. Conductive scaffolds have always served as a bioactive framework for restoring the conductive microenvironment in MI regions, thereby facilitating cell adhesion and neovascularization to the infarcted region [56]. The tissue densities of microvessels and arterioles were denser in the ECP groups, especially in the SP_0.5_-ECP group, indicating that vascularization in the infarcted area could be induced by the suitable conductive microenvironment (Fig. 8C to E). The rate of degradation may affect cellular interaction including cell proliferation, tissue synthesis, and host response [57]. The degradable property of the FSB scaffold appeared obviously beneficial for cell infiltration and further angiogenesis during the MI repairment process. In addition, the elastic modulus of the ECM surrounding the myocardium is approximately 20 to 500 kPa, which is suitable for the growth and maturation of CM [36]. Vascular endothelial cell migration and proliferation have been shown to be mediated by matrix stiffness [58]. Here, the stiffness of SP_1_ exceeded the optimum range required for the growth of CMs and endothelial cells, which in turn resulted in a relatively small number of newborn blood vessels. Based on the aforementioned results, the SP_0.5_-ECP-derived ECM-mimicking microenvironment may represent a fundamental regulatory system for enhanced endothelial cell survival and angiogenesis.

A previous study [55] has shown that the pore status in the scaffold, including pore size, porosity level, and pore interconnectivity, could considerably affect cellular biological behavior and blood vessel formation [55,59]. Furthermore, the surface topography (anisotropic microstructure) and physical/chemical cues (anisotropic mechanical–electrical properties) of exogenous scaffolds can influence cell behavior to a certain extent. Changes in roughness and electric charge trigger cell adhesion, proliferation, spread, and metabolic activity [60]. Because of these properties (appropriate pore size, porosity, elastic modulus, and conductivity), FSB-derived scaffolds can significantly inhibit cell apoptosis and promote vascular regeneration. The produced SP hydrogel exhibited directional conductivity and mechanical properties. PPy could be used to aid the alignment inside the collagen-rich fibrous matrix, which presented a uniform and ordered conductivity distribution at the microscopic scale. The CMs seeded on this anisotropic SP hydrogel exhibited elongation and achieved the correct orientation in vitro. In addition, the conductive and aligned SP-ECPs showed enhanced striated sarcomere formation compared to that associated with nonconductive and random FSB-ECPs. The conductive SP-ECPs could effectively bridge electrical conductivity between the healthy myocardium across the MI region and exhibited excellent repairing effects in vivo. Overall, the anisotropic SP hydrogel can serve as both a topographical and electroactive guide, effectively enhancing the alignment and functional maturation of cardiac muscle cells as well as for MI repair.

## Conclusion

Here, we successfully tailored the originally stiff, unstretchable, and nonconductive FSB film into a highly ordered hierarchical 3D porous hydrogel with mechanical–electrical anisotropy. The simple but elegant biorefining method endows the resultant SP hydrogel with well-aligned fiber microstructures similar to that of native cardiac tissue, showing superior biocompatibility, suitable conductivity, and biomechanical properties. Evidence from in vitro and in vivo experiments demonstrated that the natural FSB-derived conductive SP hydrogels exhibited good performance for efficiently improving the cardiac function and enhancing the electrical signal conduction and revascularization of the infarct tissues, thus facilitating the repair of infarcted cardiac tissue. Our results revealed that fish culture waste can be reused sustainably to yield a high-value cardiac patch, which may provide an alternative solution to protect the aquatic environment from pollution.

## Materials and Methods

### Materials

Py was obtained from Sigma-Aldrich Corporation (USA). Other chemical reagents were obtained from Guanghua Chemical Factory (China). Dulbecco’s modified Eagle’s medium (DMEM)/high-glucose medium, fetal bovine serum (FBS), and trypsin were purchased from Gibco (USA). The primary antibodies including CD68, Ki-67, vWF, α-smooth muscle actin (α-SMA), sarcomeric α-actinin, and CX-43 were ordered from Abcam (Britain). Alexa Fluor 488 Donkey Anti-Rabbit IgG (H+L) and Alexa Fluor 568 Donkey Anti-Mouse IgG (H+L) were procured from Life Technologies (USA).

### Preparation of the FSB film from the fish culturing waste-swim bladder

The front part of the swim bladder was collected under clean conditions, wherein the attached fat and vascular tissues on the surface were carefully removed, and then the sample was rinsed with phosphate-buffered saline (PBS; pH 7.4). This raw and hard FSB film was further cut into 20 × 20 mm^2^ pieces along the circumference and longitudinal direction, respectively, and washed thoroughly in sterile PBS. After being treated with 2.5% (m/v) NaCl solution at 4 °C overnight, the FSB was rinsed using sterile PBS several times. This resulted in a relatively soft FSB film (S).

### Preparation of the FSB/PPy hydrogel (SP) using the whole FSB film

The as-prepared FSB film (S) was transferred into NaOH solution (0.5 M) for 12 h at room temperature. It was later soaked and rinsed in 5% (m/v) H_2_O_2_ solution and shaken at room temperature for 15 min. A highly transparent and elastic FSB hydrogel was obtained accordingly. The resulting FSB hydrogel was immersed in Py solution of appropriate concentration (0.5 and 1 mg/ml) at 4 °C for 1 h, followed by soaking it completely in FeCl_3_ solution at 4 °C for 12 h to aid the polymerization of the Py monomers into PPy (noted as SP). The molar ratio of FeCl_3_ to Py monomer was 3:1. The as-prepared SP hydrogels (SP_0.5_, SP_1_) were obtained after being washed 3 times and then stored in distilled water.

### Structural characterization of FSB/PPy hydrogels

The microstructure of hydrogels was analyzed under observation by SEM (S-3000N, Hitachi, Japan). The chemical structures of the samples were verified by FT-IR (Vertex 70, Bruker, USA). XRD pattern analysis of the samples was measured using an x-ray diffractometer (Philips 1820 XRD, Shimadzu Co., Japan) with Cu-Kα radiation at 40 kV and 20 mA (5° to 45°, 2Ɵ). The thermal stability properties of the lyophilized FSB and SP hydrogels with different ratios were investigated by both DSC and TGA. DSC thermograms were obtained by using a TA DSC250 instrument. For this, the freeze-dried samples in aluminum pans were heated from 60 to 120 °C (10 °C/min) under a nitrogen atmosphere. TGA thermograms were obtained through a TG-209-Fl-Libra R instrument (Netzsch, Germany). Furthermore, the samples were heated continuously from 30 to 720 °C (10 °C/min) under a nitrogen atmosphere. High-resolution x-ray tomography analysis was carried out on a 3D x-ray microscope (XRM; Xradia Versa XRM-520, Zeiss Corp., Oberkochen, Germany) to observe the FSB hydrogel. The sample was rotated about 360°, and finally a total of 995 photos were recorded.

### In vitro degradation

The mass loss measurements over time were used to evaluate the degradation rate of the scaffolds. After being weighed, the initially dried samples were soaked into 10 ml of sterile PBS solution (pH 7.4) at 37 °C for 1 week. The redried scaffolds (*n* = 5) were weighted again, and then the corresponding weight loss was assessed. Enzymatic degradation assay of the FSB scaffolds was also investigated depending on exposure to collagenase solution (1 μg/ml) and incubated at 37 °C. At regular time intervals, the swollen scaffolds were removed from degradation solution, lyophilized, and weighted.

### Mechanical properties of the SP hydrogels along parallel and perpendicular directions

The scaffolds with a rectangular shape (length: 25.00 mm, width: 7.00 mm) were created for mechanical testing using an Instron 5542 mechanical tester. The cycling tensile test was executed up to 60% sample deformation at a compressive speed of 10 mm/min for 50 cycles. The tensile strength and anisotropic mechanical behaviors of the wet hydrogels were tested in both parallel (//) and perpendicular (⊥) directions to the aligned fiber of the swim bladder matrix in the uniaxial tension test at the same speed. At least 5 samples in each group were tested.

### Conductivity of the SP hydrogels along parallel and perpendicular directions

The conductivity of the hydrogels along the different directions (the directions parallel to the fiber and the direction perpendicular to the fiber) was evaluated using a Four-Point Probe tester (ST2258C, China). Five samples in each group were measured, and the data were analyzed through GraphPad Prism 6 and Origin Pro9 software.

### Swelling properties of the original FSB film and SP hydrogels

After the dry weights (*W*_d_) were measured, the lyophilized samples were immersed in sterile PBS buffer (pH 7.4) at 37 °C. The weights (*W*_s_) of the swollen hydrogel samples were measured at a fixed time point. The equilibrium swelling ratio (ESR) was calculated by SR = *W*_s_/*W*_d_ (3 samples/group were tested at each time point).

### The isolation and cultivation of neonatal CMs

Neonatal CMs were isolated from 1- to 3-day-old Sprague-Dawley (SD) rats according to the previously reported method [5]. The collected CMs were then seeded into the hydrogels (5 × 10^5^ per cm^2^) and cocultured at 37 °C in a humid atmosphere of 5% CO_2_ for 7 days.

### Cytoskeletal organization of the hydrogels

The samples were stained with phalloidin–rhodamine (1:40) for 40 min. The stained cytoskeleton of CMs was observed under confocal microscopy.

### Immunostaining for CM-specific markers

For detecting the level of cardiac proteins, the samples were concurrently incubated in 2 primary antibodies overnight at 4 °C, including rabbit anti-α-actinin (1:100) and mouse anti-CX-43 (1:100), and then treated with Alexa Fluor 488 donkey anti-rabbit IgG (H+L) (1:500) and Alexa Fluor 568 donkey anti-mouse IgG (H+L) (1:500) for 1 h. The stained samples were further incubated with DAPI for 1 h and imaged using laser confocal fluorescence microscopy (LCFM; LSM 880 with Airyscan, Carl Zeiss, Germany).

### Cellular alignment and distribution analysis

The elongation of CMs on aligned SP hydrogels was quantitatively measured by aspect ratio (max/min Feret diameter), which was defined as the ratio between the length of the longest line and the length of the shortest line across the nuclei [42,43]. The orientation distribution of the cells was determined from immunostaining images using the OrientationJ plugin for ImageJ software.

### Intracellular Ca^2+^ transient assays

To detect the intracellular Ca^2+^ transient of the CMs seeded on the hydrogels, all samples on day 7 of the culture were stained with the calcium indicator (50 μg of Fluo-4 AM and 50 μl of Pluronic F127 in 10 ml of PBS) for 45 min at 37 °C. The relative fluorescent intensity (*F*) profile during CM contractions (tracing the changes in Ca^2+^ concentration) was obtained by eliminating the background fluorescent intensity (*F*_0_) using ImageJ software [61].

### Electrophysiological recordings

Electrophysiology assays were performed as previously described [62]. Whole-cell patch-clamp recordings were executed at room temperature (22 to 26 °C) from the cultured CMs after 3 days of cultivation. Targeted patch-clamp recordings of the single CMs were performed under visual guidance using an ECLIPSE FN1 microscope (Nikon, Tokyo, Japan) equipped with an IR-DIC system and a Double IPA integrated patch amplifier controlled with SutterPatch software (Sutter Instrument, Novato, CA, USA). Patch pipettes with a tip resistance of 3 to 4 MΩ were filled with 130 mM K-gluconate, 15 mM KCl, 5 mM NaCl, 5 mM Mg-ATP, 1 mM MgCl_2_, 5 mM EGTA, 1 mM CaCl_2_, and 10 mM HEPES, pH 7.2. The bathing extracellular solution was of the following composition: 140 mM NaCl, 5 mM KCl, 1 mM MgCl_2_, 2 mM CaCl_2_, 10 mM HEPES, and10 mM glucose, pH 7.4. Recordings were performed using current and voltage-clamp configurations. Capacitance and input resistance were measured in voltage-clamp mode at −60 mV via a subthreshold 100-ms duration hyperpolarizing voltage step (5 mV). Spontaneous activity was observed in current-clamp configuration at the resting potential of single CMs, while induced APs, obtained by injecting suprathreshold depolarizing current pulses (0.5 nA, 5 ms), were studied while maintaining all cells at −70 mV resting potential (15 trials at 0.2 Hz for each cell). The series resistance was below 30 MΩ, and data were acquired at 10 kHz and filtered at 1 kHz.

### Beating behavior analysis

The beating behavior within CMs seeded on the hydrogels was recorded using a digital camera (E-PM2, Olympus, Japan) into 3 videos with 30-s duration time for each group, and the representative beating signals were collected and analyzed using MATLAB and ImageJ software [63].

### MI models and ECP transplantation

Animal experiments were performed under the Regulations for the Administration of Affairs Concerning Experimental Animals (China) and approved by the Southern Medical University Animal Ethics Committee. After left anterior descending (LAD) ligation for 1 week according to our previous work [4,5,64], the male SD rats (7 to 8 weeks, 250 ± 20 g) with FS < 30% were selected as MI models and randomly assigned to 5 groups, including MI group, S group, SSH group, SP_0.5_ group, and SP_1_ group. The neonatal CMs (5 × 10^5^ per cm^2^) were cocultured with the scaffolds for 7 days. Further, the CM-seeded scaffolds (ECPs) were randomly implanted into the as-prepared MI rats. The ECPs were covered onto the epicardium in the infarcted sites and fixed with 8-0 suture. In the sham group, the chests of the rats were opened to expose the hearts twice, similar to the treated groups without LAD ligation.

### LV functional analysis

One week after the MI model establishment, and 4 weeks after ECP transplantation, the left ventricular (LV) functions of the rats were evaluated using the IE33 echocardiography system (Philips Medical Systems, Nederland B.V.). M-mode and B-mode were recorded to monitor LV anterior wall morphology and beating activity. The cardiac function parameters were measured based on the 3 consecutive cardiac cycles, including LV internal dimensions at both diastole and systole (LVIDd and LVIDs), FS, and EF. All the echocardiography data were acquired and analyzed in a blinded manner by 2 investigators.

### Epicardial activation mapping

In order to examine the electrical propagation between healthy and infarcted myocardium in different groups, a 64-channel epicardial activation mapping (MappingLab) was performed on the free wall of the LV in the isolated Langendorff perfused heart. Sixty-four separate electrodes (8 × 8 grid, 0.55-mm inter-electrode distance) were placed in the boundary area between the healthy and infarcted myocardium. Electrical stimulation of 2-mV 5-Hz pulses was performed on the epicardium just below the left atrial appendage. All isochrones are generated as regions activated every 2 ms.

### Histological and immunofluorescence analysis

At 4 weeks after transplantation, the hearts were harvested and cut into frozen sections of 4-μm thickness. Myocardial fibrosis and morphology were observed by Masson's trichrome staining. Under the Masson trichrome staining, the red area indicated the myocardium and the blue area referred to collagen. The infarct size was calculated as the percentage of the scar-occupied surface area of the left ventricular anterior wall, and the wall thickness of infarct zone was averaged from 5 measurements of scar thickness. For quantification, 5 random fields in each section were counted by the same investigator in a blinded manner. These data were analyzed using Imaging Pro Plus software as previously reported [5]. To detect inflammation and myocardial repair, tissue sections were labeled with mouse anti-CD68 (1:500) rabbit anti-Ki-67 (1:250) and Tunel assay kit. For newborn vessel detection, the sections were stained with mouse anti-α-SMA (1:200) and rabbit anti-vWF (1:200). The samples were then assessed under light microscopy while focusing on the areas that have the highest numbers of capillaries and small venules (neovascular hot spots). Furthermore, the vessels were counted on 5 selected fields of the “hot spots” by the same investigator in a blinded manner.

### Statistical analysis

All the quantitative data are presented as mean ± standard deviation (*n* = 5). Differences among values were assessed using one-way analysis of variance (ANOVA) followed by the Tukey post hoc test. Comparisons between samples were evaluated by the unpaired t test. *P* < 0.05 was considered significant.

## Data Availability

The authors declare that the data supporting the findings of this study are available within the paper, and the supplementary information files.
