# Supplementary material for: Cardiac-Adaptive Conductive Hydrogel Patch Enabling Construction of Mechanical–Electrical Anisotropic Microenvironment for Heart Repair
Source: Research (Wash D C). 2023 Jun 8;6:0161. doi: 10.34133/research.0161 (PMC10250027; doi:10.34133/research.0161)
Supplement: Supplementary 2 — Figs. S1 to S23 [file research.0161.f2.docx]

Supporting Information

**Cardiac-adaptive conductive hydrogel patch enabling ECM-mimicking construction of mechanical-electrical anisotropic microenvironment for heart repair**

**Xiaoping Song^1#^, Jifeng Zhang^3#^, Si Shen^2#^, Dan Liu^2^, Wenming Yin^2^, Genlan Ye^1^, Leyu Wang^2^, Liu Cai^2^, Honghao Hou^2*^, Xiaozhong Qiu^1*^**

^1^Central Laboratory, The Fifth Affiliated Hospital, Southern Medical University, Guangzhou, Guangdong 510910, China

^2^Guangdong Provincial Key Laboratory of Construction and Detection in Tissue Engineering, School of Basic Medical Science; Biomaterials Research Center, School of Biomedical Engineering, Southern Medical University, Guangzhou, Guangdong 510515, China

^3^Department of Anatomy, Neuroscience Laboratory for Cognitive and Developmental Disorders, Medical College of Jinan University, Guangzhou, 510630, China

**^#^These authors contribute equally to this work.**

**^*^Corresponding author:**

**Prof. Honghao Hou** (ss.hhh89@hotmail.com); **Prof. Xiaozhong Qiu** (qqiuxzh@163.com)

**List of moives**

**Movie S1.** 3D X-ray microscope characterization of the FSB film

**Movie S2.** 3D X-ray microscope characterization of the Sodium chloride/Sodium hydroxide/hydrogen peroxide-treated FSB scaffold.

**Movie S3.** The beating behavior of Calcein-AM labeled cardiomyocytes seeded on the SP_0.5_ on day 7.

**Movie S4.** The elongation and orientation of cardiomyocytes seeded on the different scaffolds detected by F-actin staining.

**Movie S5.** The spontaneous beating of the different ECPs under low magnification view (10 ×) on day 7.

**Movie S6.** The spontaneous beating of the different ECPs under low magnification view (10 ×) on day 14.

**Movie S7.** Video recording of calcium signal propagation in the different ECPs under low magnification view (10 ×).

**Movie S8.** Echocardiograph videos in rats after the different ECPs transplantation for 4 weeks.

**Supplemental Results**

**
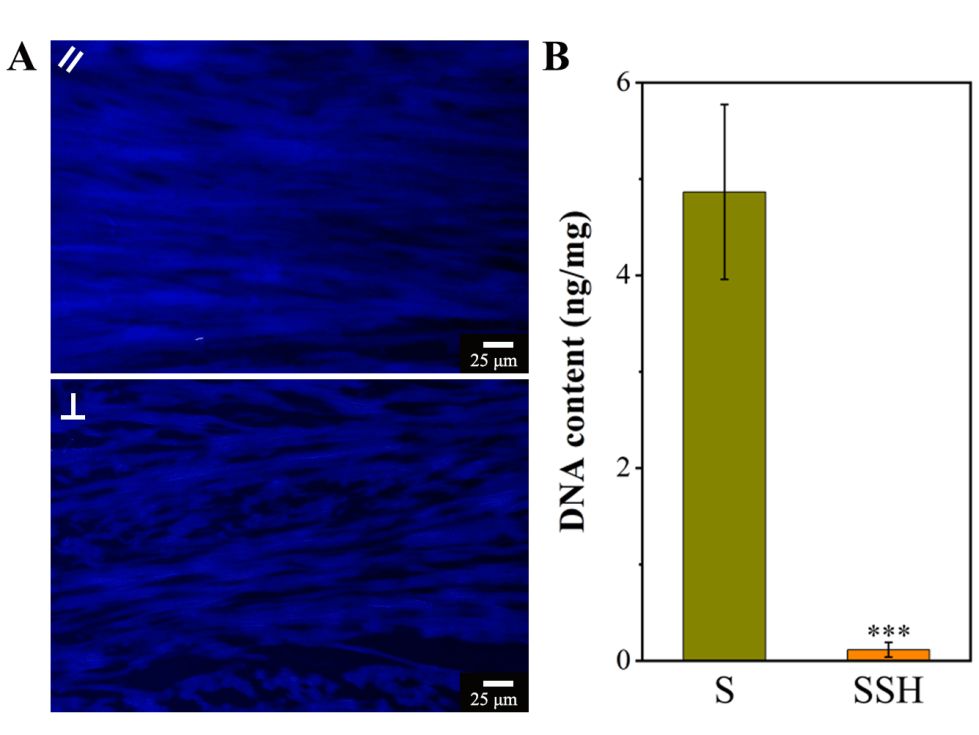
**

**Fig. S1. Detection of residual cell nuclei in the FSB scaffold after treatment. (A)** DAPI staining of SSH scaffolds after processing. **(B)** Residue DNA before (S) and after decellularization (SSH). n=5. ^***^ compared to the untreated S, p < 0.001.


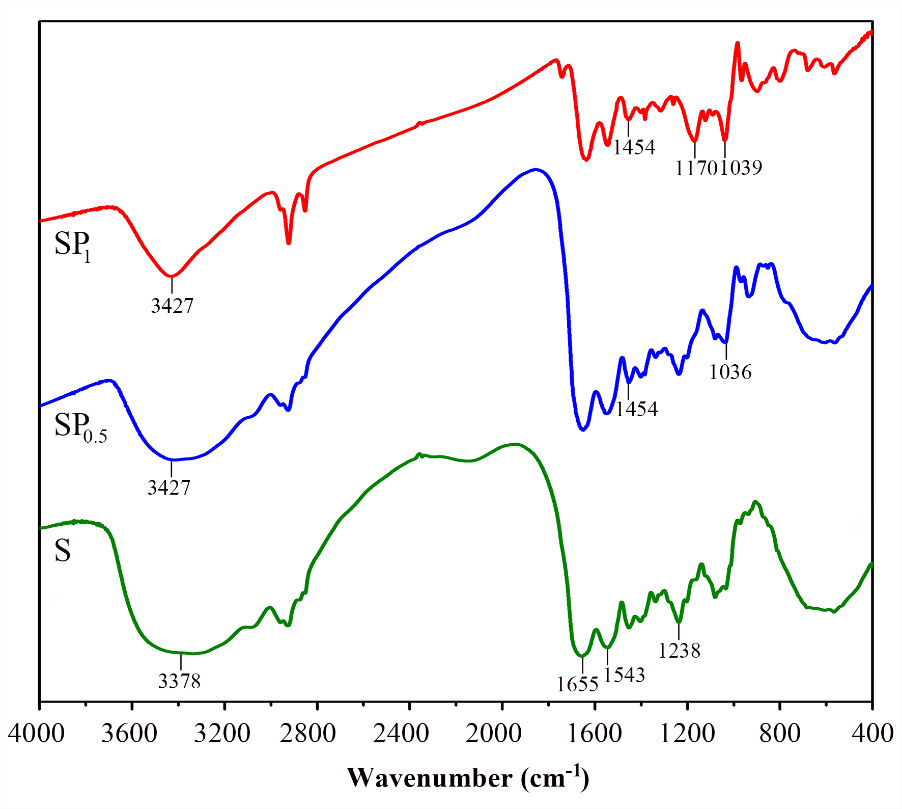


**Fig. S2. FT-IR spectra of the different FSB-derived scaffolds.**


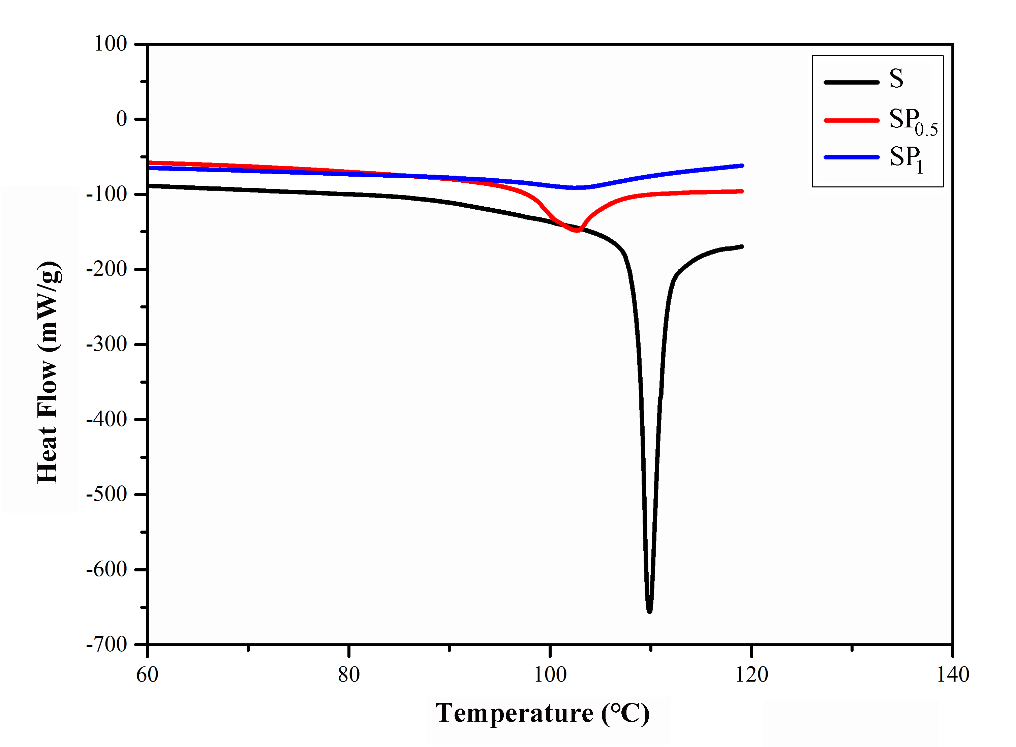


**Fig. S3. Differential calorimetry scanning (DSC) analysis of the different scaffolds.**


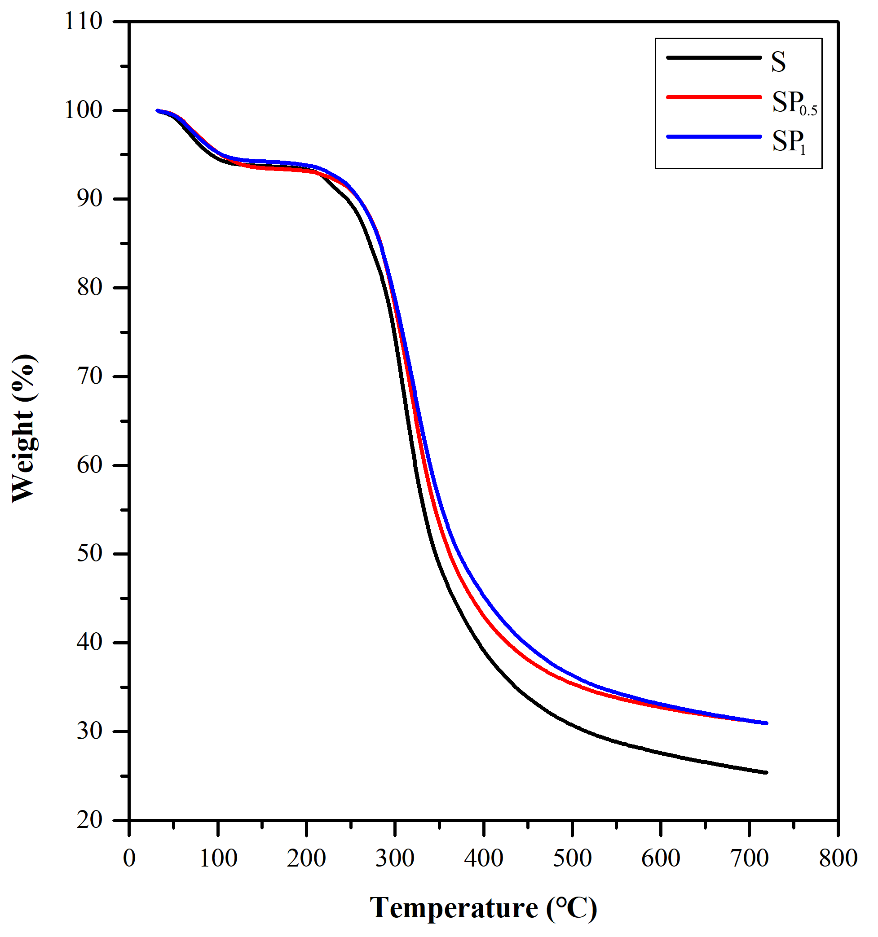


**Fig. S4. Thermogravimetric analysis (TGA) curves of the different scaffolds.**


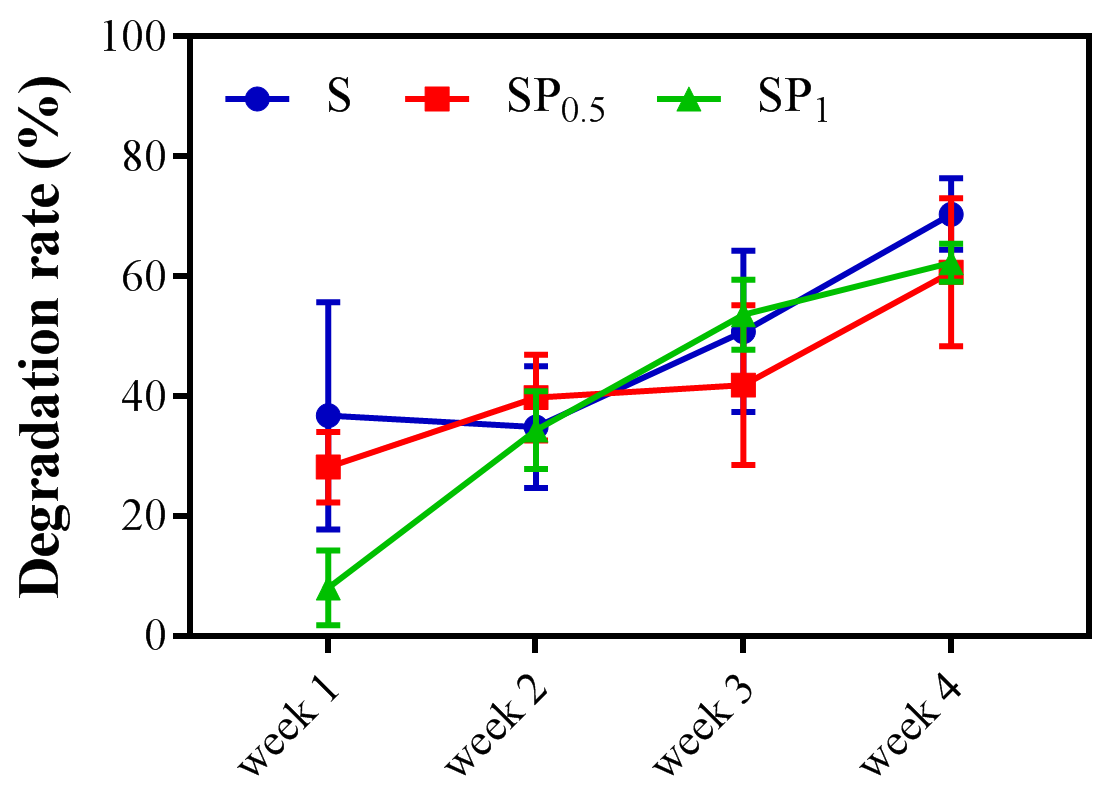


**Fig. S5. Degradation curves of the different scaffolds.** n=5.


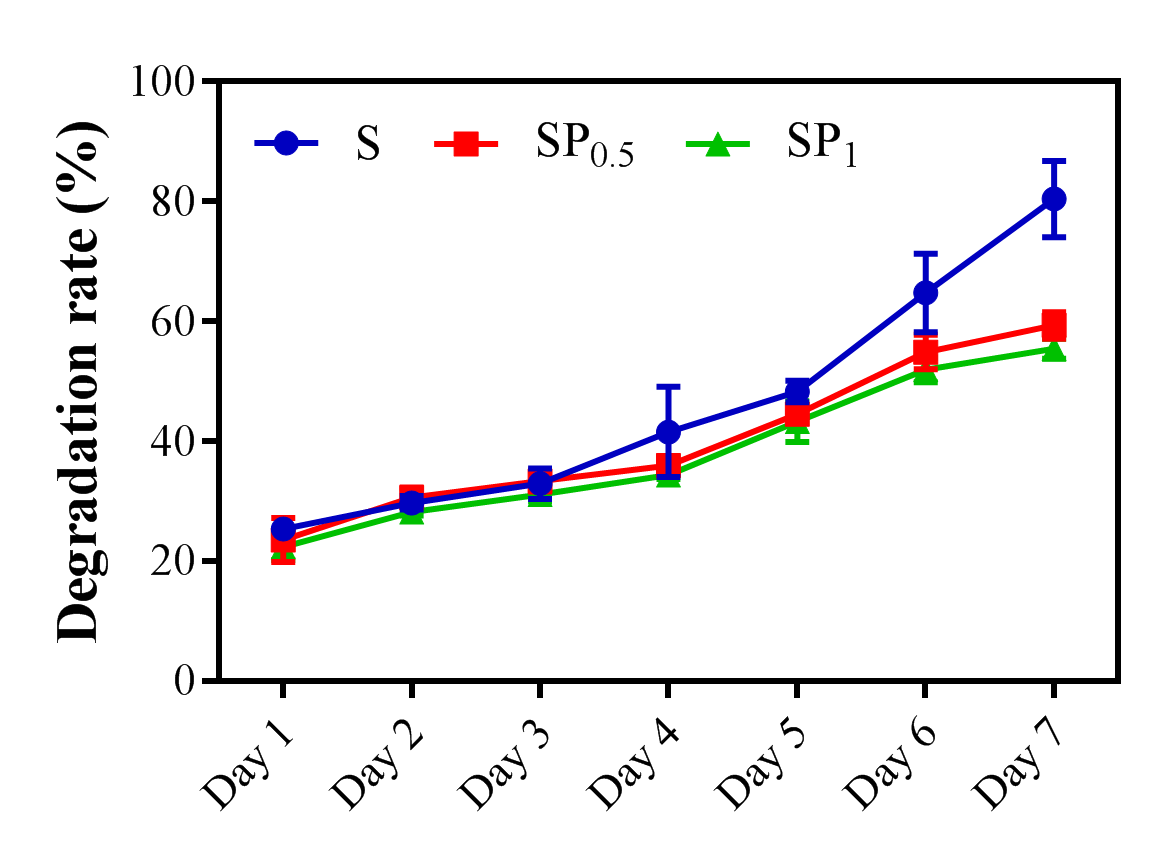


**Fig. S6. The bio-degradation degree of the three kinds of FSB scaffolds after incubation in collagenase solution for different time periods.** n=5.


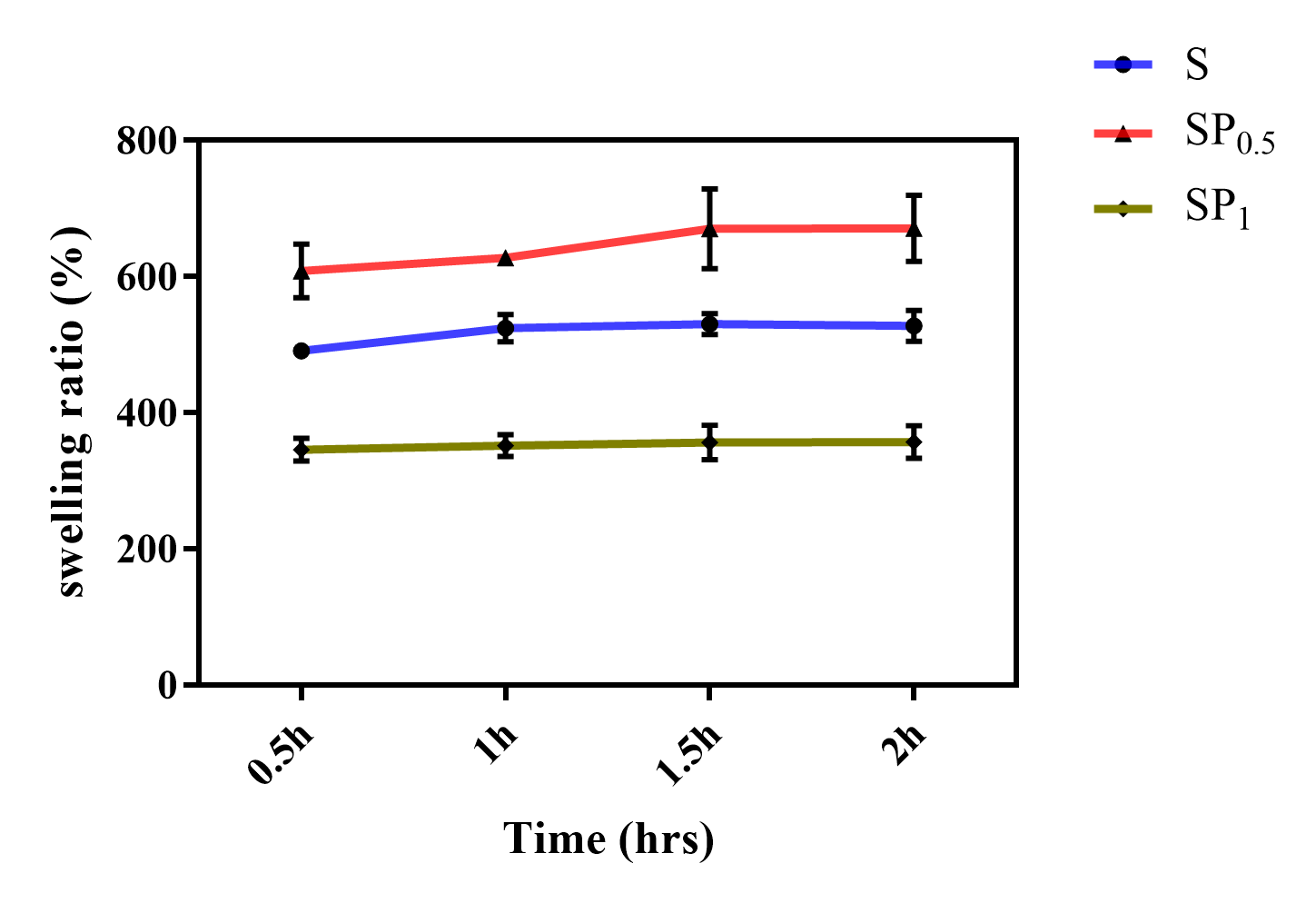


**Fig. S7. Swelling ratio of different scaffolds at different time points.** n=5.


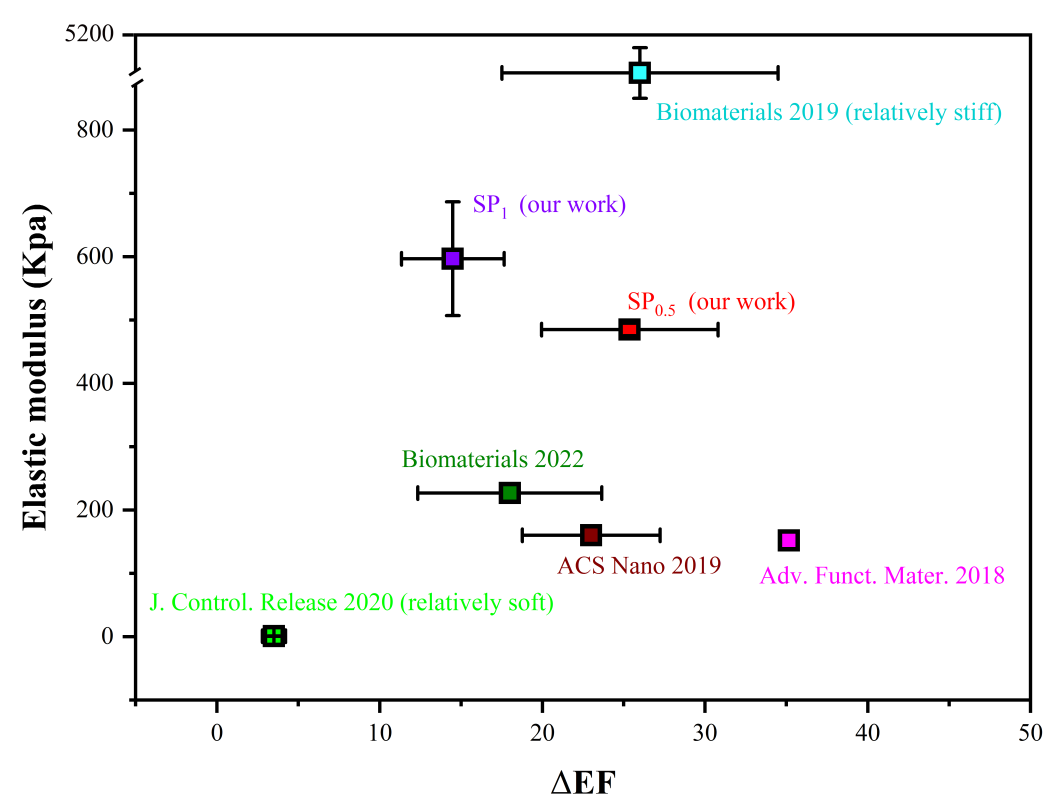


**Fig. S8. Comparison of elastic modulus and ejection fraction of different scaffolds.**


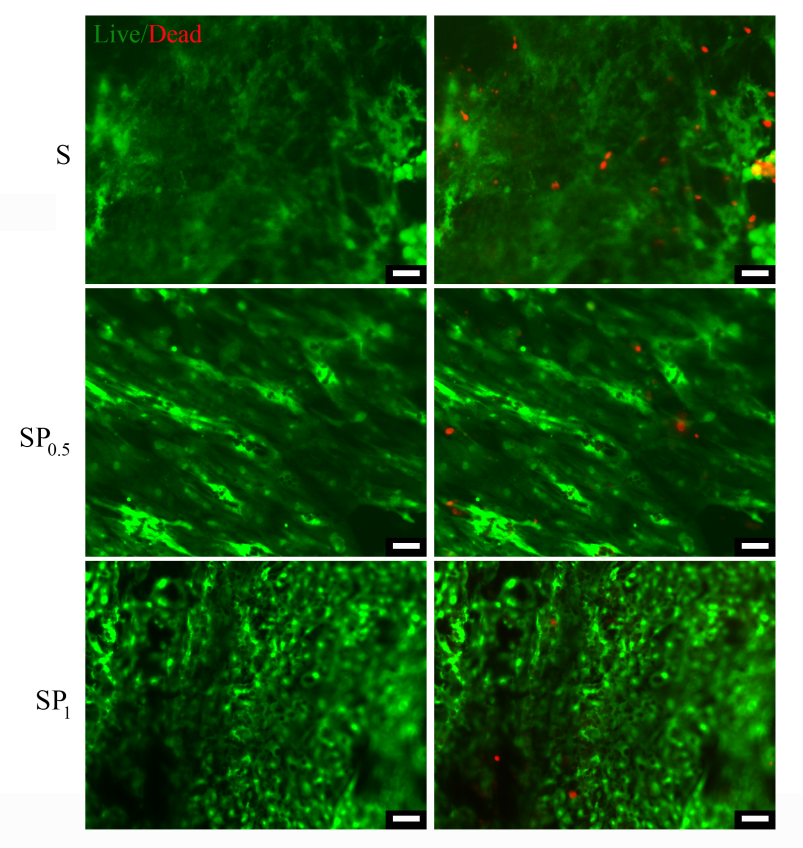


**Fig. S9. The growth status of cardiomyocytes on the scaffolds after 21 days of culture. Scale bars, 50 μm.**


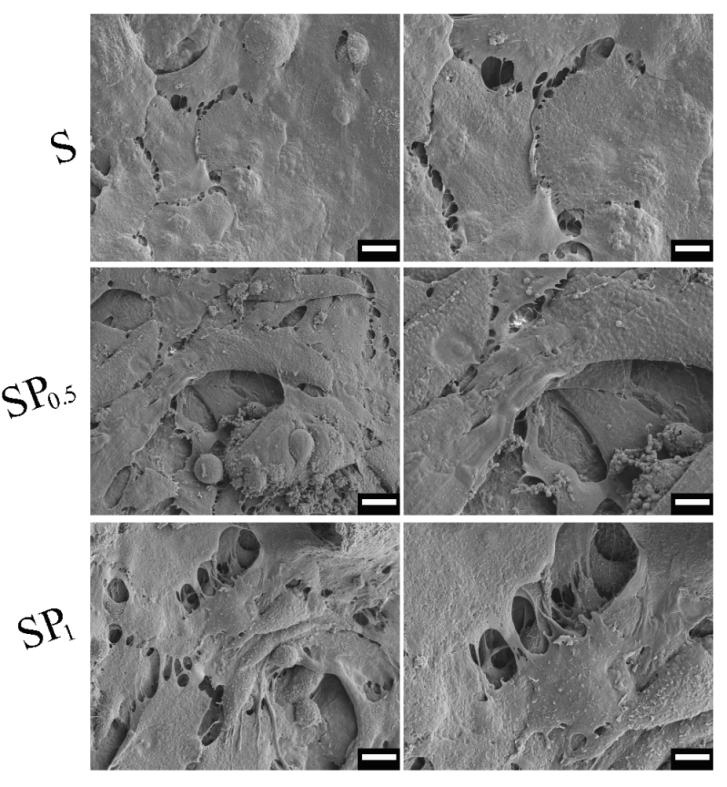


**Fig. S10. Corresponding SEM images of Cardiomyocytes on the different scaffolds.** Scale bars: left: 10 μm; right: 5 μm.

**
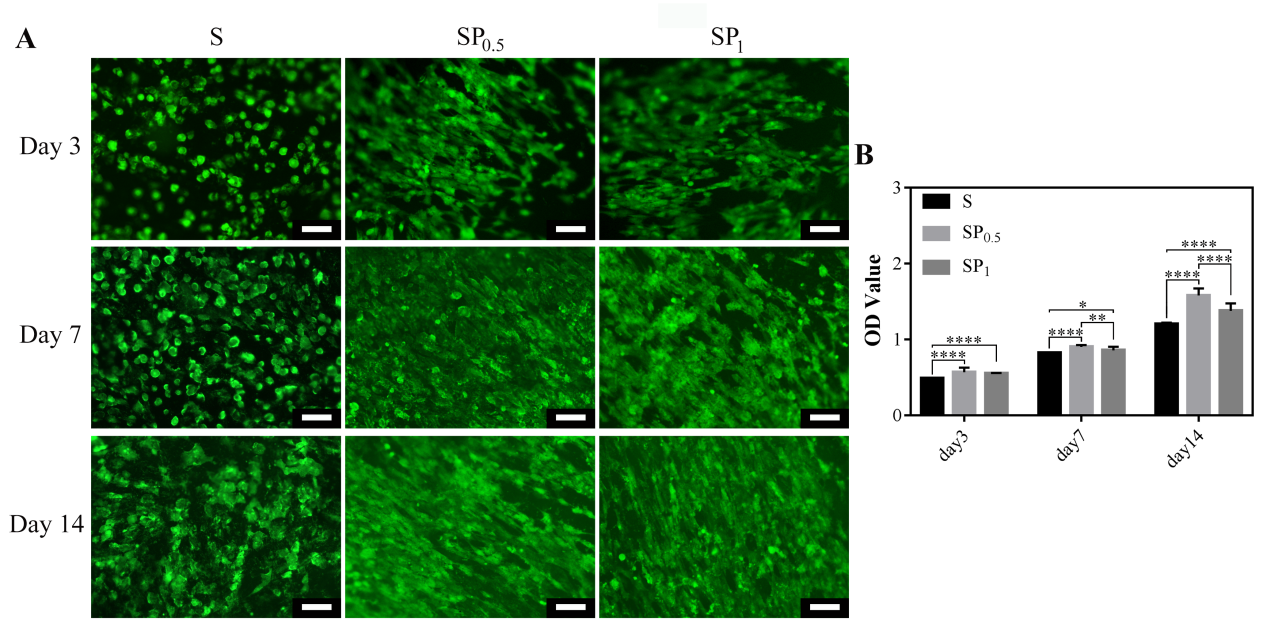
**

**Fig. S11. The growth status of H9C2 in different time periods on each group. (A)** The live/dead staining of H9C2 in each group. Scale bars, 100 μm. **(B)** The cell viability in different time periods on each group via CCK-8 testing. n = 5. ^*^p < 0.05, ^**^p < 0.01, ^****^p < 0.0001.


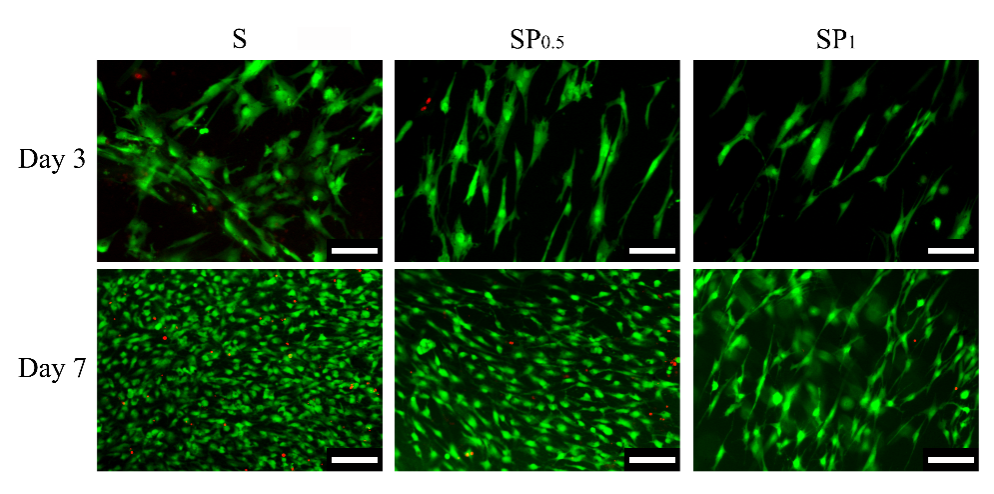


**Fig. S12. The growth status of cardiac fibroblast on each group at Day 3 and Day 7.** Scale bars: 100 μm.


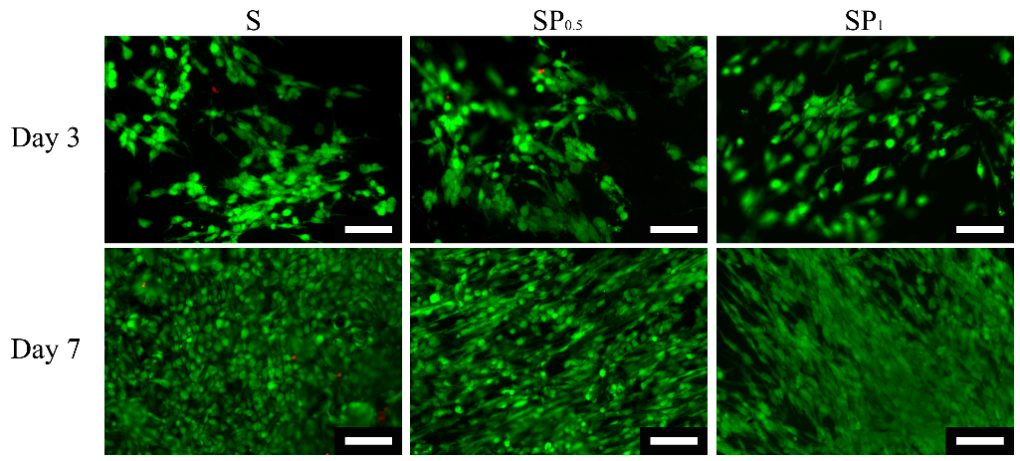


**Fig. S13. The growth status of HUVEC on each group at Day 3 and Day 7.** Scale bars: 100 μm.


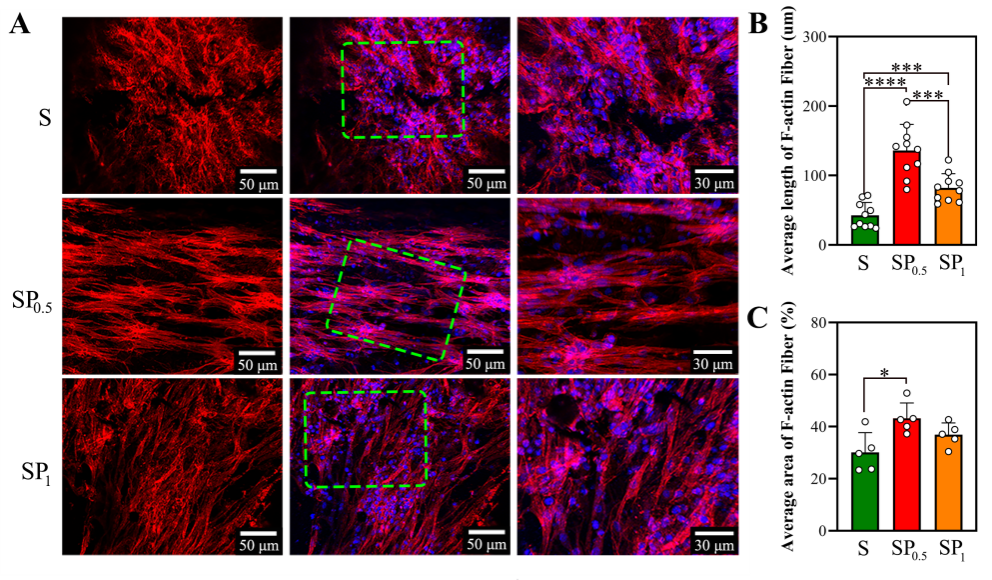


**Fig. S14. Anisotropic SP hydrogels induced cardiomyocytes alignment. (A)** 3D Fluorescence microscopy images of cells cultured on three kinds of scaffolds at day 7, with F-actin stained with Rhodamine-phalloidin (red) and nuclei with DAPI (blue). **(B)** Average fiber length in different scaffolds. **(C)** Average fiber area in different scaffolds.


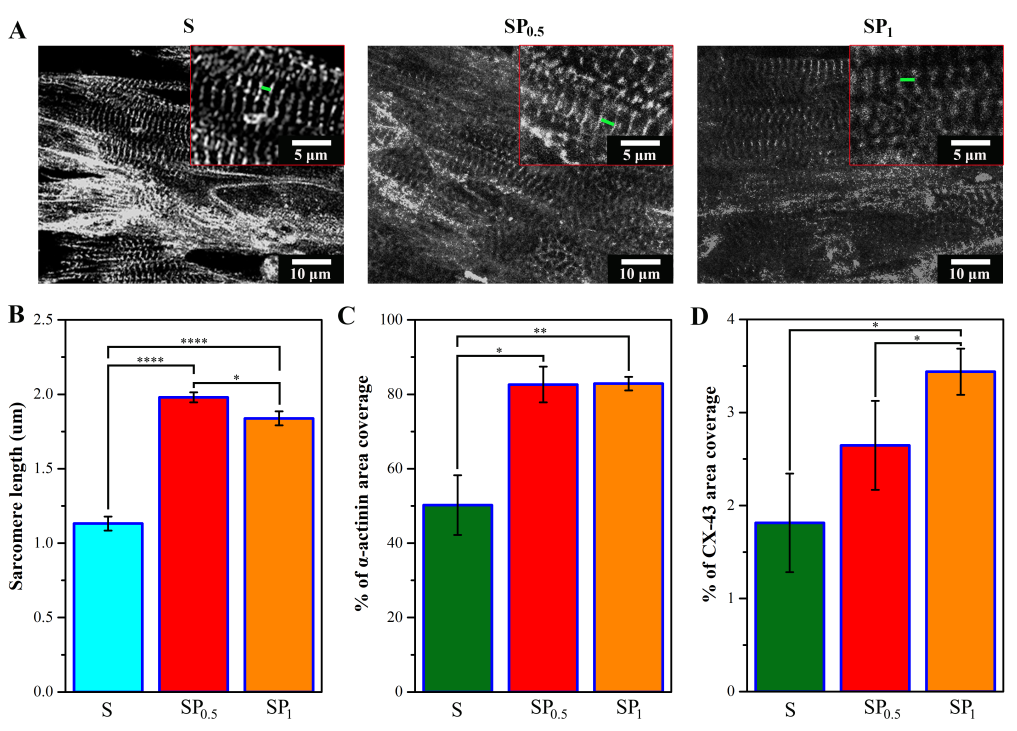


**Fig. S15. The apparent cardiomyocyte elongation was observed on the conductive SP hydrogel. (A) Comparison of sarcomeres in the three groups of materials. (B-D)** The statistical analysis concerning sarcomere length, α-actinin and CX-43 coverage on the different scaffolds. n=3. ^*^ p < 0.05; ^****^ p < 0.0001.


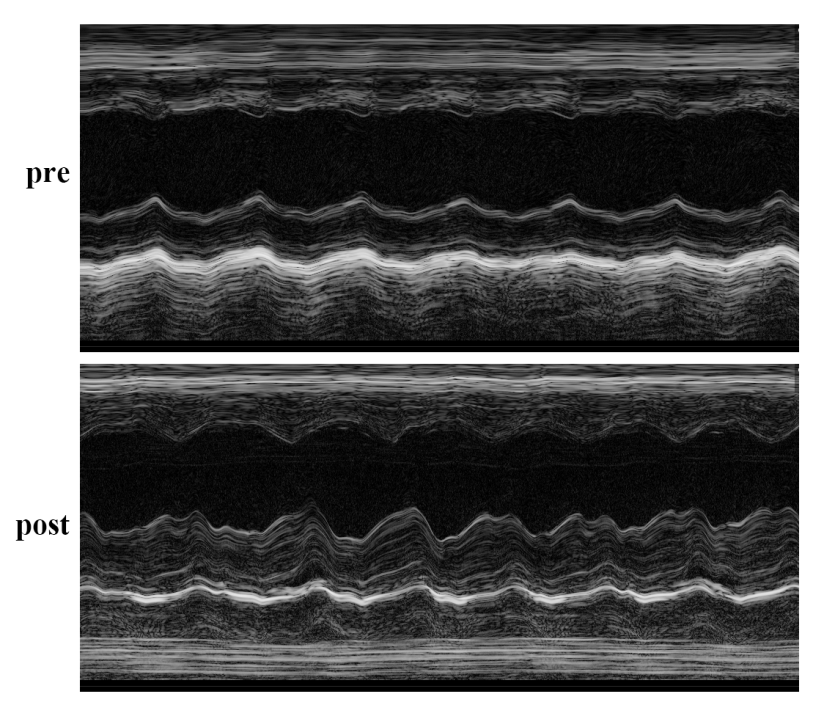


# Fig. S16. Echocardiographic images for hearts in the sham group before and after 4 weeks.


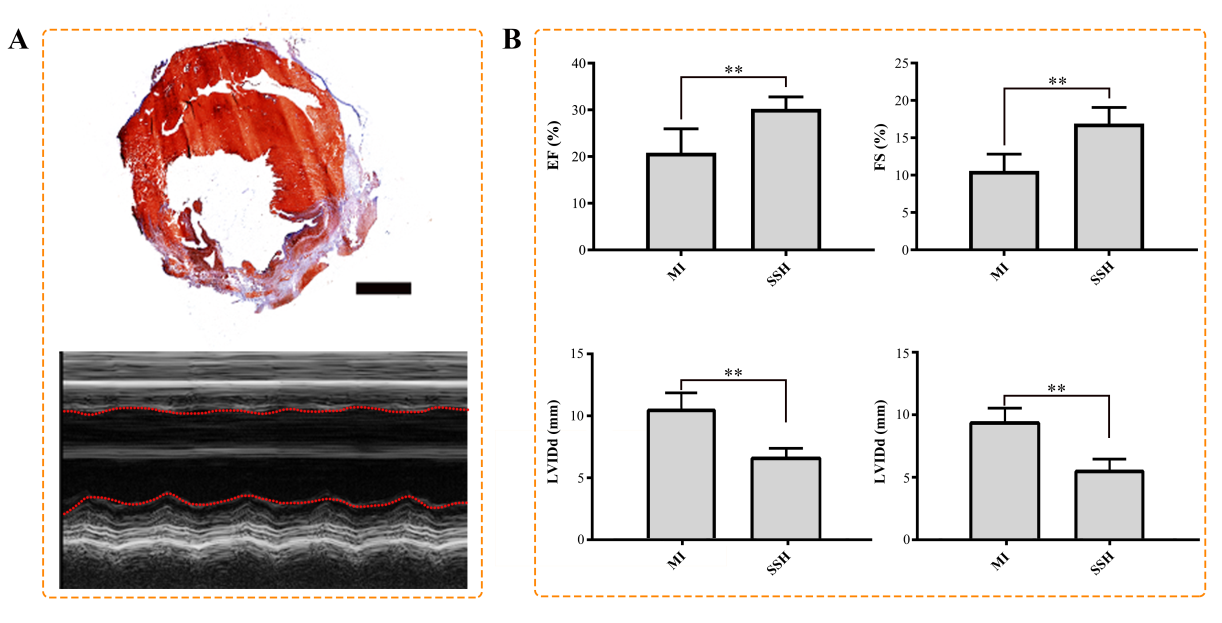


**Fig. S17. The effects of the SSH ECP on MI repair . (A) Representative echocardiographic and Masson's trichrome staining images for hearts.** Scale bars = 2.5 mm. **(B)** **The left ventricular function parameters (EF, FS, LVIDd, and LVIDs) in the SSH group after implantation for 4 weeks.** n = 5. **p < 0.01.


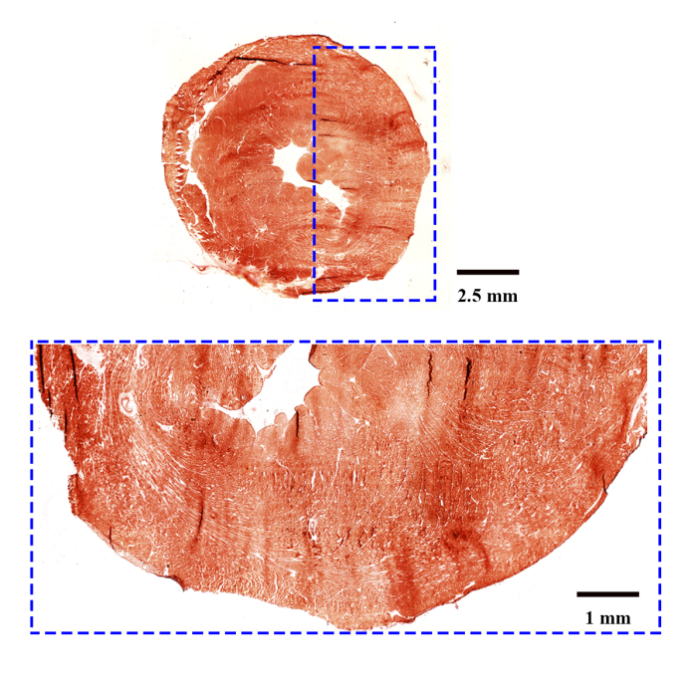


# Fig. S18. Representative Masson's trichrome staining in the sham group after 4 weeks.


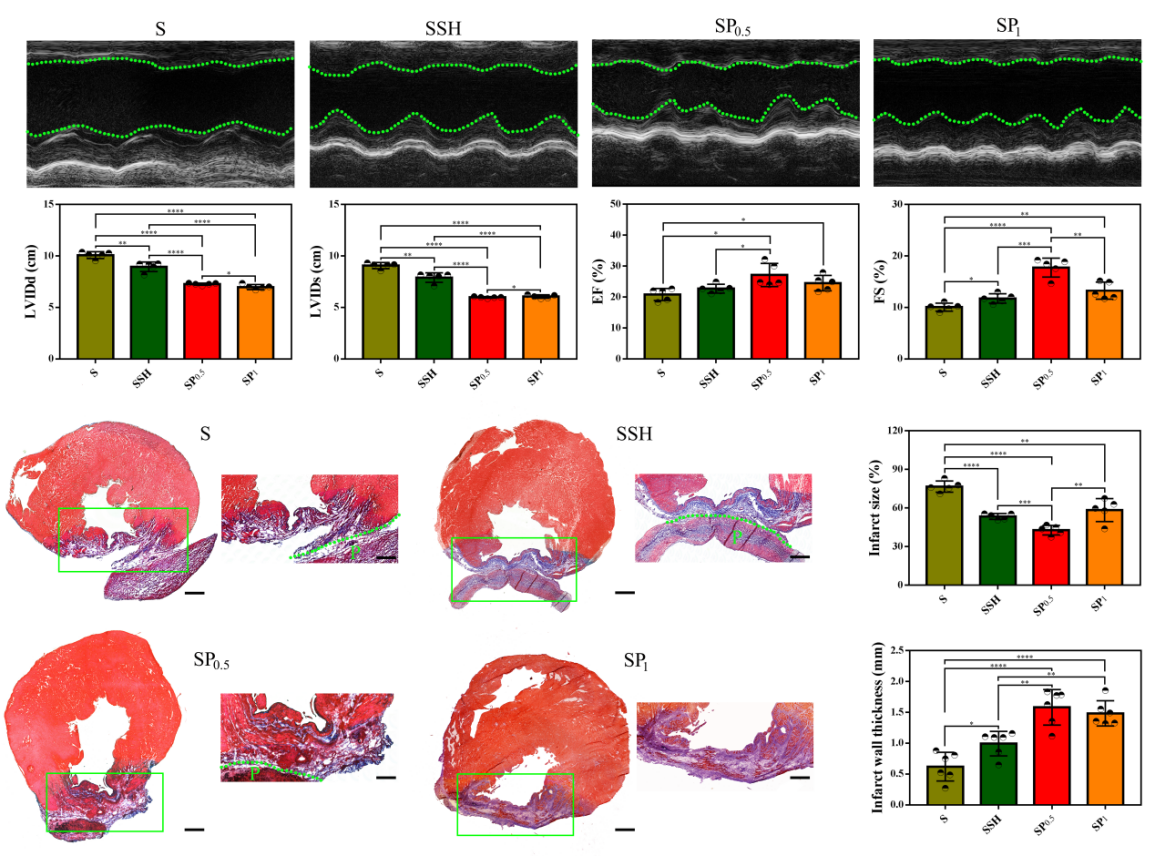


**Fig. S19. *in-vivo* Myocardial infarction repair studies with different empty scaffolds implantation for 4 weeks in a rat MI model.** Scale bars: left in each image, 1mm; right in each image, 500 μm.

**
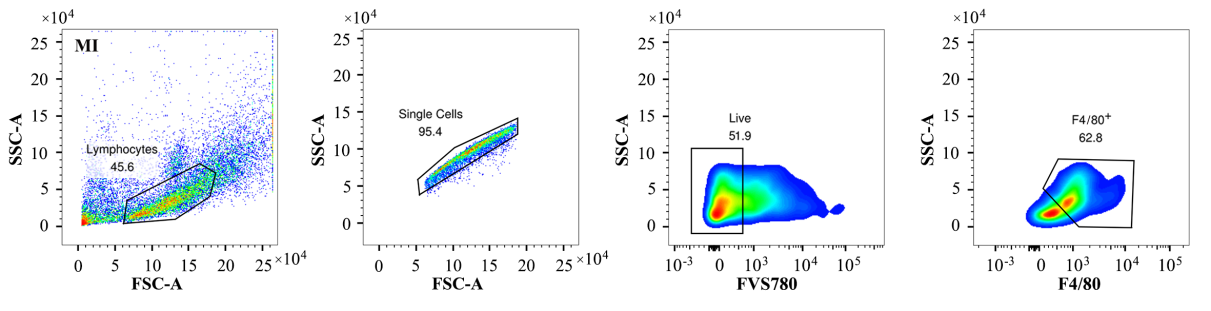
**

**Fig. S20. The flow cytometry results one week after transplantation in the MI hearts.**


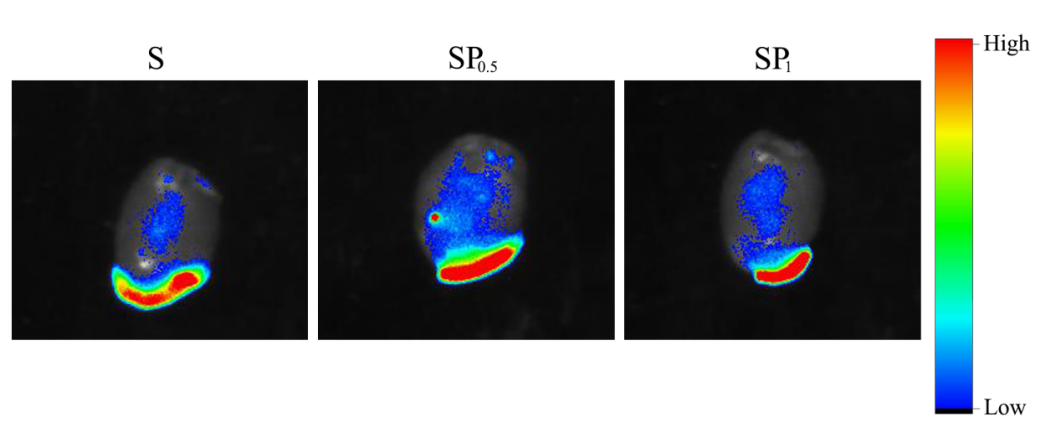


**Fig. S21. Fluorescence images of MI hearts indicated obvious cardiomyocyte retention in the MI zone after scaffolds transplantation on day 7.**


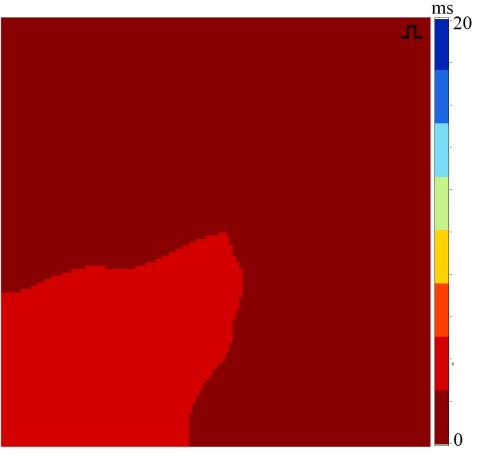


**Fig. S22. Electrical map was recorded from the rats in the sham group.**


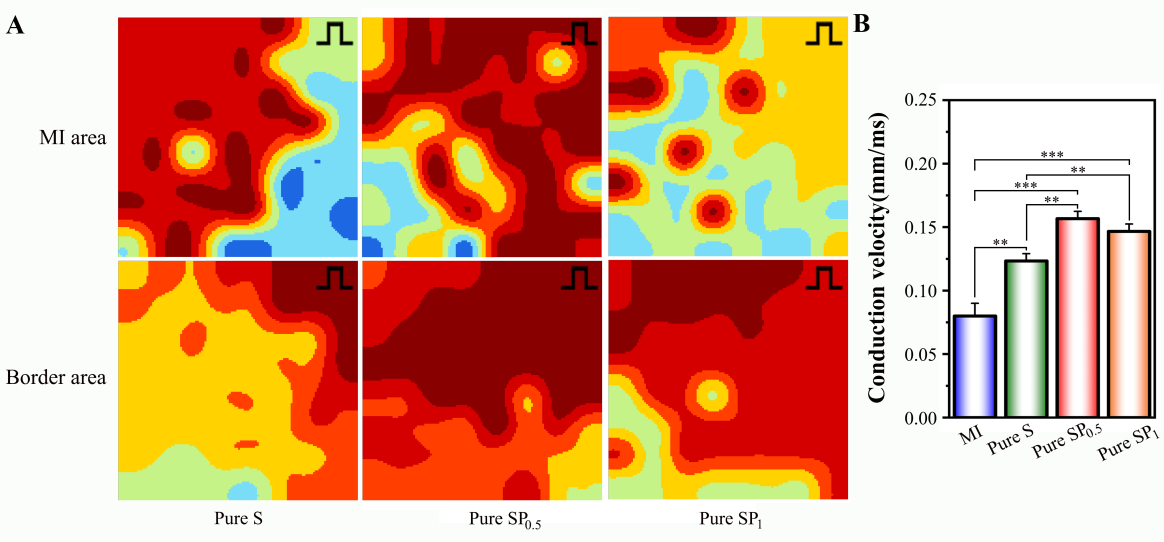


**Fig. S23.** **The electrical conduction in the junction area of different material transplantation groups. (A)** Representative electrical maps were recorded from the rats in the different pure scaffold-transplant groups. **(B)** The conduction velocity between infarcted and healthy sites in the pure scaffold-transplant groups. n=5. ^**^p < 0.01; ^***^p < 0.001.
